# Putative role of TMEM165 in congenital cardiomyopathies

**DOI:** 10.3389/fnmol.2025.1692968

**Published:** 2026-01-09

**Authors:** Paula P. Gonçalves

**Affiliations:** CESAM – Centre for Environmental and Marine Studies, Department of Biology, University of Aveiro, Aveiro, Portugal

**Keywords:** TMEM165 (human transmembrane protein 165), Ca^2+^/H ^+^ antiport, cardiomyocytes, heart, disorders of glycosylation, congenital cardiomyopathies

## Abstract

Within the significant worldwide causes of mortality and morbidity are congenital heart diseases. Congenital cardiomyopathies include conditions in which early diagnosis and care can improve survival and health. In general, the first diagnostic tool is clinician suspicion followed by appropriate imaging, classically an echocardiogram. Cardiomyopathies have high rates of clinically detectable genetic causes. In view of this, prompt genetic testing is highly recommended for patients with cardiomyopathy. Genetic diagnosis, that is relevant to both the patient and family members, can help guide the selection of appropriate therapies and provide valuable information about the presence of comorbidities in other organ systems. Congenital Disorders of Glycosylation (CDG) are a growing group of inherited multisystem disorders characterized by defects in the glycosylation of proteins and lipids. Hypertrophic / dilated cardiomyopathy and neuromuscular abnormalities are recurrent manifestations of glycosylation defects. Mutations within the gene encoding the human transmembrane protein 165 (HsTMEM165), that belong to uncharacterized protein family 0016 (UPF0016), have been associated with cases of CDG. Recent progress in basic and clinical research related to TMEM165, focusing on the pathogenicity of HsTMEM165 variants, are reviewed. Highlights include the critical role of amino acid replacement for maintaining the structural and functional integrity of TMEM165 and their known associations with phenotypes of CDG patients. Future directions in this rapidly evolving area of research are proposed, to recognize the potential involvement of HsTMEM165 in congenital cardiomyopathies.

## Introduction

Glycolysis and glycosylation are of paramount importance in the context of congenital disorders of glycosylation (CDG) and cardiomyopathies. Glycolysis supplies ATP and intermediates that intersect with glycan metabolism. Interconversion of *α*-D-glucose1-phosphate (G1P) and α-D-glucose 6-phosphate (G6P) links glycolysis, glycogen metabolism, the pentose phosphate pathway and galactose utilization. By shaping uridine diphosphate-sugar pools, it secondarily impacts protein glycosylation. Glycosylation is one of the most versatile and diverse post-translational modifications of proteins, in which oligosaccharide moieties (glycans) are covalently attached to proteins or lipids. Glycan chains are covalent linked to glycosylation sites of proteins. Namely to asparagine residues (*N*-glycosylation) and/or to serine/threonine/tyrosine/hydroxylysine (*O*-glycosylation), and/or to tryptophan (*C*-mannosylation) (Steentoft et al., 2013; Marques-da-Silva et al., 2017). Its intrinsic processes, in general terms, begin with the initial transfer of glycosyl in the endoplasmic reticulum (ER), followed by its entry into the Golgi complex, where a variety of glycans are added to facilitate glycan maturation, involving a panoply of enzyme activities (Schjoldager et al., 2020; He et al., 2024).

**Figure 1:**
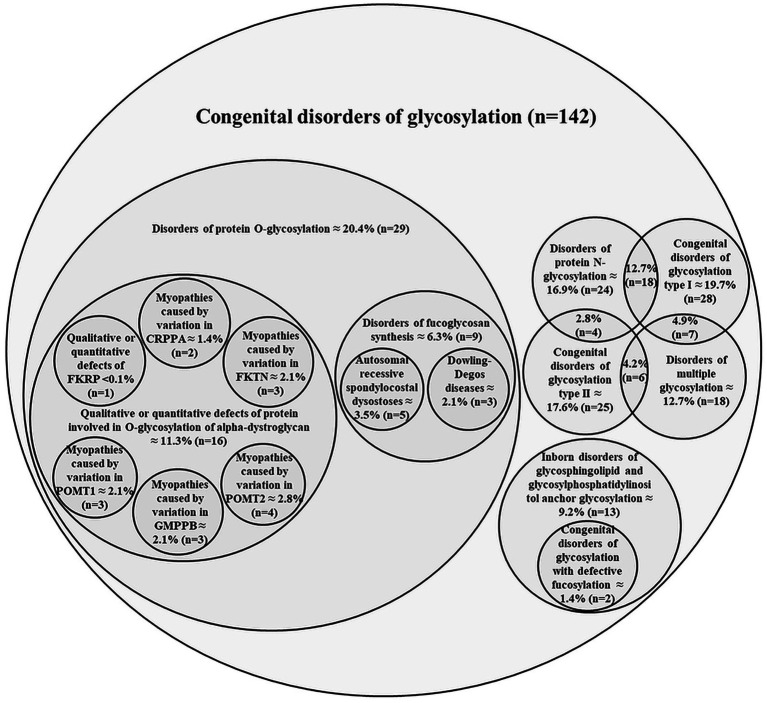
Illustrative representation of the hierarchical classification of congenital disorders of glycosylation for which causal genes have been identified. The numbers represent the percentage contributions of each CDG sub-class. In parentheses is the total number of sub-classes included. Data from The Monarch Initiative database (Putman et al., 2024). Explanation in the text.

In 2024, more than 200 distinct types of CDG were identified, caused by defects in nearly 190 different genes (Ng et al., 2024). These diseases encompass multiple categories of glycosylation pathways (e.g., *N*-linked glycosylation, *O*-linked glycosylation, lipid glycosylation, trafficking defects, etc.), reflecting the breadth of glycan-related biology. Illustrative representation of the hierarchical classification of congenital disorders of glycosylation for which causal genes have been identified is shown in Figure 1 and Supplementary Table S1. Disorders of *N*-glycosylation can be subdivided into CDG-I (defects in the assembly of the dolichylpyrophosphate linked oligosaccharides and/or their transfer to asparagine residues on the nascent polypeptides in cytosol and endoplasmic reticulum) and CDG-II [abnormal *N*-glycan processing mostly at the level of the Golgi apparatus including defects in enzymes and transporters involved in glycosylation, vesicular trafficking and pH homeostasis (e.g., vesicular H^+^-ATPase subunit ATP6V0A and COG complex)].

**Figure 2:**
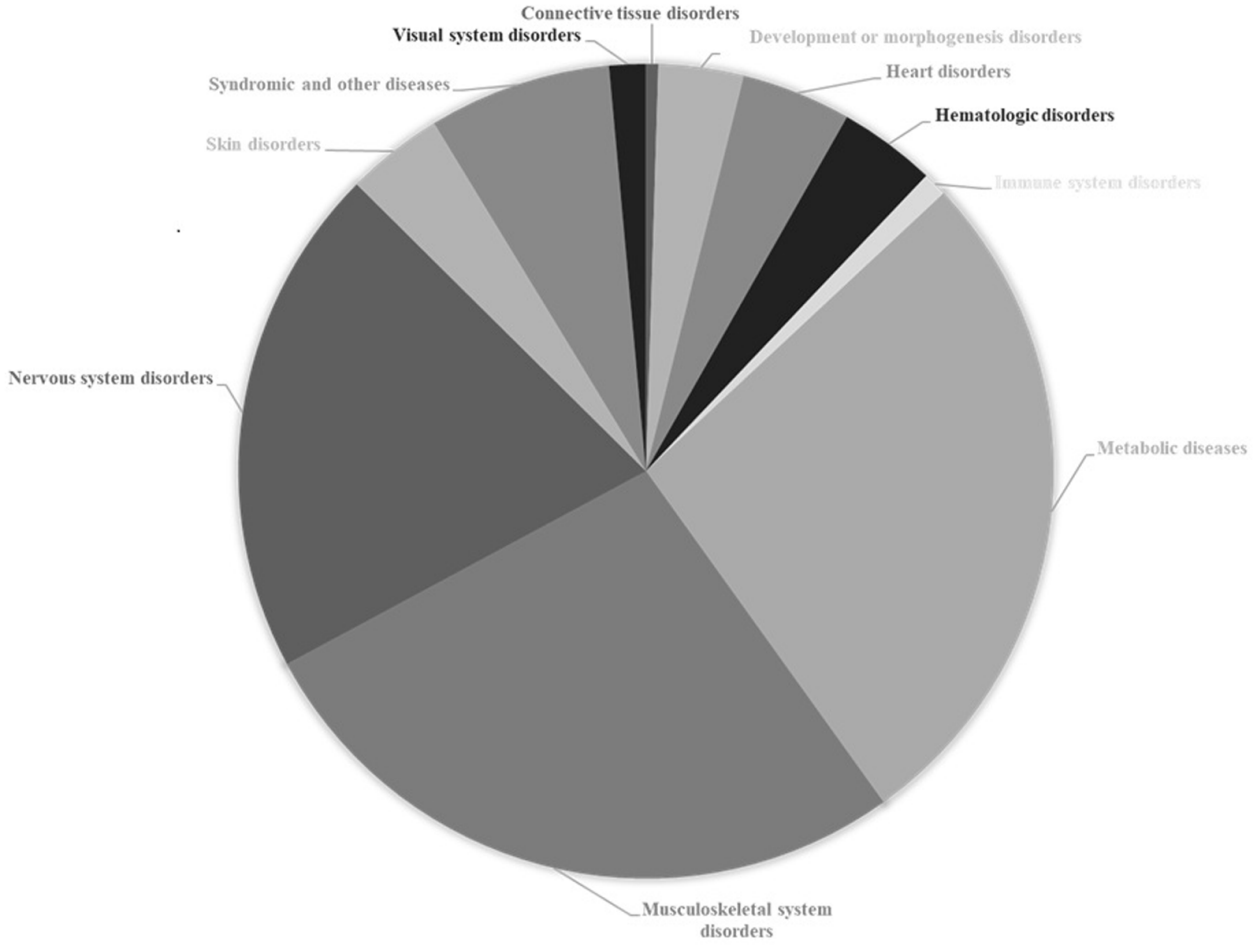
Association of causal genes of congenital disorders of glycosylation with other pathological conditions. Data from The Monarch Initiative database (Putman et al., 2024). Explanation in the text.

One of the most prominent characteristics of CDG is the multiplicity of symptoms, whose clinical evaluation is indicative of the simultaneous dysfunction of several organ systems (Figure 2). In addition to syndromic, metabolic and other diseases that account for 34% of diseases associated with genes causing glycosylation disorders, musculoskeletal and nervous system disorders are by far the most relevant, representing 45% of the total diseases associated with these genes. Each of the other disorders (heart, hematologic, skin, visual system, immune system, connective tissue, development or morphogenesis disorders) individually contribute percentages of less than 5%.

Heart defects interfering with blood flow through the heart and its body circulation are a common type of birth defect and represent a major global health challenge. Congenital heart defects account for more than 200,000 deaths worldwide per year. Cardiac development and function are tightly coupled to energy metabolism, membrane integrity and extracellular matrix glycoprotein dynamics. Therefore, defects in glycolysis and glycosylation can result in congenital heart diseases and cardiomyopathies. The hypertrophic and dilated cardiomyopathies, major causes of congestive heart failure, are illustrative of this paradigm. Hypertrophic cardiomyopathy, characterized by left ventricular hypertrophy, is a frequent inherited cardiac condition that often is detected only when a sudden cardiac death event occurs. Elevated levels of aldolase fructose-bisphosphate A, complement C3, glutathione S-transferase omega 1, Ras suppressor protein 1, talin 1, and thrombospondin 1 in the circulating plasma of patients with hypertrophic cardiomyopathy are well-correlated with cardiovascular magnetic resonance imaging markers (wall thickness of left ventricle, mass, and percentage myocardial scar) of phenotype severity (Captur et al., 2020). Every so often dilated cardiomyopathy is associated with right ventricular disease, and it is characterized by left ventricular dilation, systolic dysfunction and secondary diastolic dysfunction due to cardiac enlargement. Besides its heterogeneous etiology (drug/alcohol abuse/toxicity, myocarditis, as well as metabolic and ischemia-induced abnormalities), approximately a quarter of cases correspond to a familial disease. In familial dilated cardiomyopathies abnormal function of cytoskeletal/contractile proteins points mostly to altered cytoarchitecture of cardiomyocytes and their structural connections (Arimura et al., 2013), but also to the glycolytic enzymes and mitochondrial impairment, reflecting the energy-intensive workload adaptation of cardiomyocytes. Several genetic alterations have been identified as directly linked to this pathological condition, leading to mutated proteins, namely actin, desmin, dystrophin, lamin A/C, LIM domain-binding protein 3 (LDB3), four and a half LIM domains protein 2 (FHL2), myosin, phospholamban, phosphoglucomutase 1, sarcoglycans, titin, troponin T, tropomyosin and vinculin.

Many other types of cardiomyopathies have been associated with disturbances in carbohydrate metabolism and glycosylation (Conte et al., 2023). In addition to clinical conditions resulting from the loss of the heart muscle’s ability to pump blood, congenital heart diseases are also caused by anatomical defects of the heart and other less frequent situations such as cutis laxa and arterial tortuosity. More broadly, disturbances in carbohydrate metabolism and glycosylation are recurrently associated with diverse cardiomyopathies and structural heart defects. Congenital heart defects and cardiomyopathies are also associated abnormalities reported in some CGD patients (Oberstein et al., 2006; Reis et al., 2008; Dassie-Ajdid et al., 2009; Haldeman-Englert et al., 2009; Shimizu et al., 2010; Faletra et al., 2011; Marques-da-Silva et al., 2017).

**Figure 3:**
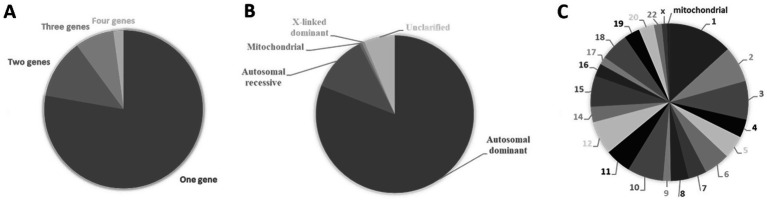
Causal genes of congenital heart diseases. **(A)** Percentage of congenital heart diseases caused by variations within a single gene (monogenic) or multiple genes (polygenic). **(B)** Types of inheritance of congenital heart diseases expressed in percentage. **(C)** Percentual distribution of causal genes according to their cytogenetic location. Data from The Monarch Initiative database (Putman et al., 2024). Explanation in the text.

The known number of genes that cause congenital heart diseases is constantly growing Figure 3 and Supplementary Table S2 detail inheritance patterns and chromosomal distribution. These diseases are mostly monogenic (Figure 3A) with autosomal dominant inheritance (Figure 3B). The causal genes can be found in almost all chromosomes but are not uniformly distributed (Figure 3C). Eventually, clustering of genes in specific regions of chromosomes 1, 2, 3, 6, 10, 11, 12, 15 and 18 may occur (Figure 3C; Supplementary Table S2). Recently, congenital heart disease risk loci were identified, e.g., PGM1, MACROD2, GOSR2, WNT3 and MSX1 that play an essential functional role in heart development at the embryonic and newborn stages emphasizing their role in pathways central to embryonic cardiac development (Lahm et al., 2021).

A particular set of other glycolysis/glycosylation-related genes deserves attention, namely B3GLCT, B3GAT3, CHST3, MGAT2, PIGL, DOLK and COG1. These genes encode enzymes involved in various glycosylation pathways, and mutations in each are known to cause congenital disorders (including CDG subtypes or other syndromes) that often feature structural heart defects or cardiomyopathy (see Table 1; Supplementary Table S2 for an overview of each gene and its associated disorder). Next, these examples are highlighted to emphasize how defects in glycosylation pathways can lead to cardiac pathology.

**Table 1.** Selected causal genes of congenic cardiac diseases by separated systematic searches performed for each disorder, gene, protein and metabolic pathway.

Causal Gene	B3GLCT	B3GAT3	CHST3	COG1	DOLK	MGAT2	PIGL	PGM1
HGNC ID	HGNC:20207	HGNC:923	HGNC:1971	HGNC:6545	HGNC:23406	HGNC:7045	HGNC:8966	HGNC:8905
Chromosome Location	13q12.3	11q12.3	10q22.1	17q25.1	9q34.11	14q21.3	17p11.2	1p31.3
Protein Name	beta 3-glucosyltransferase	beta-1,3-glucuronyltransferase 3	carbohydrate sulfotransferase 3	component of oligomeric Golgi complex 1	dolichol kinase	alpha-1,6-mannosyl-glycoprotein 2-beta-*N*-acetylglucosaminyltransferase	phosphatidylinositol glycan anchor biosynthesis class L	phosphoglucomutase 1
Ion Requirements		Mn^2+^, Zn^2+^, Co^2+^, Cu^2+^ or Mg^2+^	Ba^2+^, Ca^2+^, Co^2+^, Cu^2+^, Mg^2+^, Mn^2+^, Sr^2+^ or Zn^2+^			Mn^2+^		Mg^2+^
Subcellular Distribution	Endoplasmic reticulum	Single-pass type II membrane protein of Golgi apparatus membrane and *cis*-Golgi network	Single-pass type II membrane protein of Golgi apparatus membrane	Peripheral membrane protein of Golgi apparatus membrane	Multi-pass membrane protein of endoplasmic reticulum membrane	Single-pass type II membrane protein of Golgi apparatus membrane	Single-pass type I membrane protein of endoplasmic reticulum membrane	Cytoplasm
Disease	Peters plus syndrome	Larsen-like syndrome, B3GAT3 type	Spondyloepiphyseal dysplasia with congenital joint dislocations	COG1-congenital disorder of glycosylation	DK1-congenital disorder of glycosylation	MGAT2-congenital disorder of glycosylation	CHIME syndrome	PGM1-congenital disorder of glycosylation
MONDO ID	MONDO:0009856	MONDO:0009511	MONDO:0007738	MONDO:0012637	MONDO:0012556	MONDO:0008908	MONDO:0010221	MONDO:0013968
Inheritance	Autosomal recessive	Autosomal recessive	Autosomal recessive	Autosomal recessive	Autosomal recessive	Autosomal recessive	Autosomal recessive	Autosomal recessive
Biochemical Process								
HALLMARK GLYCOLYSIS (M5937)		**+**						
HALLMARK HYPOXIA (M5891)			**+**					**+**
HALLMARK ADIPOGENESIS (M5905)								**+**
PID HIF 1 TF PATHWAY (M255)								**+**
HALLMARK MTORC1 SIGNALING (M5924)								**+**
carbohydrate metabolism (GO:0005975)	**+**	**+**	**+**			**+**		
galactose metabolism (GO:0006012)								**+**
galactose catabolism (GO:0019388)								**+**
galactose catabolism via UDP-galactose (GO:0033499)								**+**
GPI anchor metabolism (GO:0006505)					**+**		**+**	**+**
fucose metabolism (GO:0006004)	**+**							
nucleotide-sugar metabolism (GO:0009225)	**+**							**+**
amino sugar metabolism (GO:0006040)			**+**					**+**
*N*-acetylglucosamine metabolism (GO:0006044)			**+**					
dermatan sulfate proteoglycan metabolism (GO:0050655)		**+**						
carbohydrate biosynthesis (GO:0016051)						**+**		**+**
oligosaccharide biosynthesis (GO:0009312)						**+**		
oligosaccharide biosynthesis (GO:0009312)								
other types of *O*-glycan biosynthesis (hsa00514)	**+**							
chondroitin sulphate biosynthesis (GO:0030206)			**+**					
chondroitin sulfate proteoglycan biosynthesis (GO:0050650)		**+**	**+**					
synthesis of substrates in *N*-glycan biosynthesis (R-HSA-446219)					**+**			**+**
dolichyl monophosphate biosynthesis (GO:0043048)					**+**			
dolichol phosphate mannose biosynthesis (GO:0180047)					**+**			
dolichyl diphosphate biosynthesis (GO:0006489)					**+**			
proteoglycan biosynthesis (WP4784)			**+**					**+**
GPI anchored protein biosynthesis (GO:0180046)							**+**	
GPI anchor biosynthesis (GO:0006506)							**+**	
dermatan sulfate proteoglycan biosynthesis (GO:0050651)		**+**						
protein *O*-linked fucosylation (GO:0036066)	**+**							
fucosylation (GO:0036065)	**+**							
protein *O*-linked glycosylation (GO:0006493)		**+**			**+**			
protein *N*-linked glycosylation via asparagine (GO:0018279)						**+**		
glycosylation (GO:0070085)	**+**	**+**	**+**	**+**	**+**	**+**	**+**	**+**
Golgi organization (GO:0007030)				**+**				
intra-Golgi vesicle-mediated transport (GO:0006891)				**+**				
retrograde (vesicle recycling within Golgi) transport (GO:0000301)				**+**				
viral protein processing (GO:0019082)						**+**		

Phosphoglucomutase 1 is encoded by the PGM1 gene, mutations of which underlie the PGM1-CDG. The PGM1 protein is a central factor in multiple signaling and metabolic pathways that may produce different biochemical pathomechanisms and, consequently, distinct phenotypes in patients (Beamer, 2021). Phosphoglucomutase 1, which catalyzes the bi-directional interconversion between G1P and G6P, is a key enzyme in glycolysis, glycogenolysis and glyconeogenesis and pentose phosphate pathway. This enzyme also impacts protein glycosylation, since it is linked to galactose metabolism (Conte et al., 2020; Backe et al., 2020). Its deficiency causes PGM1-CDG, with multisystem phenotypes (Morava, 2014; Tegtmeyer et al., 2014; Wong et al., 2016). Clinical manifestations include midline facial defects, bifid uvula, growth retardation, hepatopathy, malignant hyperthermia, hypogonadotropic hypogonadism, hypothyreoidism, hyperinsulinism, hypoglycemia, myopathy, hemostatic anomalies and heart anomalies (enlarged heart, ventricular septal defect, aorta coarctation, cardiomegaly, early-onset dilated cardiomyopathy). Dilated cardiomyopathy, one of the most clinically important phenotypes associated with PGM1 defects, often leads to cardiac arrest and untimely demise (Wong et al., 2016). This feature appears to be explained by jeopardized phosphoglucomutase 1 binding to the heart-muscle-cell–specific splice variant of Z band alternately spliced PDZ-containing protein (ZASP) (Arimura et al., 2009; Tegtmeyer et al., 2014; Wong et al., 2016). PGM1 interaction with the Z-disk protein ZASP provides a structural link to cardiomyopathy.

The Peters plus syndrome, a rare autosomal recessive disease, is caused by defects in B3GLCT that encodes for a *β*-1,3-glucosyltransferase. This enzyme, which transfers glucose to *O*-linked fucosylglycans on proteins, is a type II membrane protein of the endoplasmic reticulum. This syndrome is recurrently characterized by developmental defects with reported cardiac defects, and variable phenotypes that include corneal defects, short limbs, characteristic facial features, mild to severe intellectual disability, genitourinary system disorders and hypothyroidism (Oberstein et al., 2006; Reis et al., 2008; Dassie-Ajdid et al., 2009; Haldeman-Englert et al., 2009; Shimizu et al., 2010; Faletra et al., 2011; Weh et al., 2014; Marques-da-Silva et al., 2017).

B3GAT3 and CHST3 are the genes that encode for β-1,3-glucuronyltransferase 3 (EC 2.4.1.135) and carbohydrate sulfotransferase 3 (EC 2.8.2.17), two single-pass type II membrane proteins of Golgi apparatus. The *β*-1,3-glucuronyltransferase 3 catalyzes the final step in the biosynthesis of the linkage region of proteoglycans. This involves the formation of a glycosaminoglycan-protein linkage through a glucuronyl transfer reaction and requires Mn^2+^ for full activation. The carbohydrate sulfotransferase 3 is essential for sulfation of chondroitin, an important proteoglycan for cell migration and differentiation. B3GAT3 and CHST3 are causal genes of Larsen-like syndrome, a primary bone dysplasia characterized by laxity, dislocations and contractures of the joints, short stature, hand and foot malformations, short neck, craniofacial dysmorphism, ocular defects and various cardiac presentations (Tuysuz et al., 2009; Baasanjav et al., 2011; Von Oettingen et al., 2014; Jones et al., 2015; Job et al., 2016; Bloor et al., 2017; Marques-da-Silva et al., 2017; Ranza et al., 2017; Yauy et al., 2018; Ritelli et al., 2019; Li et al., 2022).

Another Mn^2+^ requiring hexosyltransferase of the Golgi apparatus membrane is the *α*-1,6-mannosyl-glycoprotein 2-β-*N*-acetylglucosaminyltransferase (EC 2.4.1.143), which is responsible for converting oligomannose to complex *N*-glycans. Mutations in MGAT2 gene can lead to MGAT2-CDG, also known as carbohydrate-deficient glycoprotein syndrome, type II. This disorder affects the glycosylation process, leading to abnormal glycoprotein production and a range of symptoms. Profound global developmental disability, hypotonia, early onset epilepsy, facial dysmorphism and other multisystem manifestations, including liver dysfunction, bleeding tendency and arrhythmogenic disorders are also reported (Poskanzer et al., 2021).

Mutations in PIGL gene are recognized as causing the Coloboma–congenital heart disease–ichthyosiform dermatitis–mental retardation– ear anomalies (CHIME) syndrome. The loss of function of PIGL enzyme (EC 3.5.1.89), a single-pass type I membrane protein of endoplasmic reticulum, disrupts the second step of glycosylphosphatidylinositol synthesis (de-*N*-acetylation of *N*-acetylglucosaminylphosphatidylinositol), which results in the disruption of protein glycosylation. As the name of the genetic disorder indicates, in addition to colobomas, migratory ichthyosiform dermatosis, mental retardation, ear anomalies, cardiac dysfunction are some of its main symptoms (Shashi et al., 1995; Tinschert et al., 1996; Ng et al., 2012; Johnson et al., 2017; Marques-da-Silva et al., 2017).

Another integral membrane enzyme of endoplasmic reticulum, the dolichol kinase (EC 2.7.1.108), which is encoded by the DOLK gene, is central to phosphorylation of dolichol, a glycosyl carrier lipid essential for various glycosylation processes, namely *C*- and *O*-mannosylation, *N*- and *O*-linked glycosylation of proteins, and biosynthesis of glycosyl phosphatidylinositol anchors. DK1-CDG, which is caused by homozygous or heterozygous compound mutations in the DOLK gene, is mainly characterized by developmental delays, seizures, other neurological symptoms, muscular hypotonia, ichthyosis and cardiac defects (Kranz et al., 2007a; Kranz et al., 2007b; Lefeber et al., 2011; Kapusta et al., 2013; Lieu et al., 2013; Marques-da-Silva et al., 2017; Komlosi et al., 2021; Yu et al., 2022).

Finally, *N*- and *O*-glycosylation defects are repeatedly attributed to protein dysfunctionality of the eight subunits of the Golgi Oligomeric Complex (GOC), which is essential for glycosylation enzymes, maintenance of Golgi structure, and regulation of membrane protein trafficking within the Golgi and for retrograde transport from the Golgi to the endoplasmic reticulum (D'Souza et al., 2020). COG1 is the gene that encodes the COG1 component of the Oligomeric Golgi Complex 1, a protein that is part of the conserved oligomeric Golgi complex (COG), which is essential for the normal structure and function of the Golgi apparatus. This complex participates in glycosylation and vesicular transport. Mutations of COG1 leads to COG1-CDG, causing facial dysmorphism, developmental delays, intellectual disability, seizures and other health problems, reflecting abnormal hepatic, gastrointestinal, skeletal and cardiac functioning (Wu et al., 2004; Spaapen et al., 2005; Foulquier et al., 2006; Morava et al., 2007; Zeevaert et al., 2009; Marques-da-Silva et al., 2017; Quelhas et al., 2021; Salazar et al., 2021). Congenital cardiac anomalies associated with mutations in the subunits of Oligomeric Golgi Complex cause early mortality in severe multisystem diseases. COG mutations lead to the COG-CDG, another severe multi-systemic disease. Among the most common clinical presentations are perinatal asphyxia, hyperkeratosis, developmental disability, hypotonia, severe microcephaly, dysmorphia, skin abnormalities, psychomotor and growth retardation, skeletal deformities, microcephaly, recurrent epilepsies, hepatosplenomegaly, gastrointestinal pseudo-obstruction, renal and cardiac abnormalities (Tegtmeyer et al., 2014; D'Souza et al., 2020). During the first years of life, a high mortality rate due to congenital cardiac defects (patent ductus arteriosus, dysplastic aortic valve and atrial and ventricular septal defects) appears to be especially related not only to mutated COG1, but also COG6 and COG7 (Morava et al., 2007; Rymen et al., 2015; D’Souza et al., 2021).

These and other glycolysis/glycosylation genes converge on structural valve anomalies, septal defects, aorta and pulmonary artery malposition, arrhythmias, and cardiomyopathies (hypoplastic left ventricle, hypertrophic and dilated forms, including severe biventricular dilation). The diversity of cardiac phenotypes is illustrated in Table 1 and Supplementary Table S2 by showing that the proteins encoded by the above highlighted genes, participate in a variety of metabolic and cellular regulatory pathways, of which glycosylation is shared by all. Hence, genetic mutations in these genes disrupt glycosylation and so cause CDG, a group of multisystemic diseases with high clinical heterogeneity. Interestingly, these different monogenic diseases are all linked to cardiac defects. Cardiac manifestations ranging from heart and valvular defects (tetralogy of Fallot), cardiomegaly with heart right deviation, transposition of the great arteries, double outlet ventricle, absence of right pulmonary vein, dilated aortic root, patent/persistent ductus arteriosus and foramen oval, atrial and ventricular septal defects, valve anomalies (stenosis, prolapse and regurgitation), arrhythmogenic disorders (tachycardia, bradycardia, arrhythmia, episodic asystole and acute congestive heart failure) and cardiomyopathies (hypoplastic left ventricle, and hypertrophic and dilated cardiomyopathies, including severe biventricular dilation) were described in the literature (Wu et al., 2004; Oberstein et al., 2006; Morava et al., 2007; Reis et al., 2008; Dassie-Ajdid et al., 2009; Haldeman-Englert et al., 2009; Zeevaert et al., 2009; Shimizu et al., 2010; Baasanjav et al., 2011; Faletra et al., 2011; Von Oettingen et al., 2014; Weh et al., 2014; Jones et al., 2015; Job et al., 2016; Wong et al., 2016; Bloor et al., 2017; Marques-da-Silva et al., 2017; Yauy et al., 2018; Li et al., 2022).

Beyond the genes above mentioned, SRD5A3 and ALG1/6/8 deserve attention due to cardiac findings, including severe early onset of multisystem diseases with cardiac involvement. Patients with different CDG (e.g., SRD5A3-CDG, as well as ALG1-, ALG6-, and ALG8-CDG) may have cardiac symptoms besides the ones previously mentioned (Gehrmann et al., 2003; Morava et al., 2008; Vesela et al., 2009; Morava et al., 2012; Morava et al., 2016; Ben Ayed et al., 2021). The autosomal recessive SRD5A3-CDG (MONDO:0012885) is caused by mutations in the SRD5A3 gene on chromosome 4q12. Steroid 5*α*-reductase 3 (EC 1.3.1.94), a multi-pass membrane protein of endoplasmic reticulum, catalyzes the conversion of polyprenol into dolichol, required for the synthesis of dolichol-linked monosaccharides and the oligosaccharide precursor used for *N*-glycosylation (Ben Ayed et al., 2021). Chitobiosyldiphosphodolichol *β*-mannosyltransferase (EC 2.4.1.142), dolichyl-P-Glc: Man9GlcNAc2-PP-dolichol α-1,3-glucosyltransferase (EC 2.4.1.267) and dolichyl-P-Glc: Glc1Man9GlcNAc2-PP-dolichol α-1,3-glucosyltransferase (EC 2.4.1.265) are hexosyltransferases encoded by the genes ALG1 (16p13.3), AlG6 (1p31.3) and ALG8 (11q14.1), respectively. Loss-of-function mutations in these genes can cause severe forms of autosomal recessive congenital disorders of *N*-linked glycosylation (MONDO:0012052, MONDO:0011291 and MONDO:0011969), by blocking protein glycosylation steps in the endoplasmic reticulum (Vesela et al., 2009; Morava et al., 2012; Morava et al., 2016; Captur et al., 2020).

**Figure 4:**
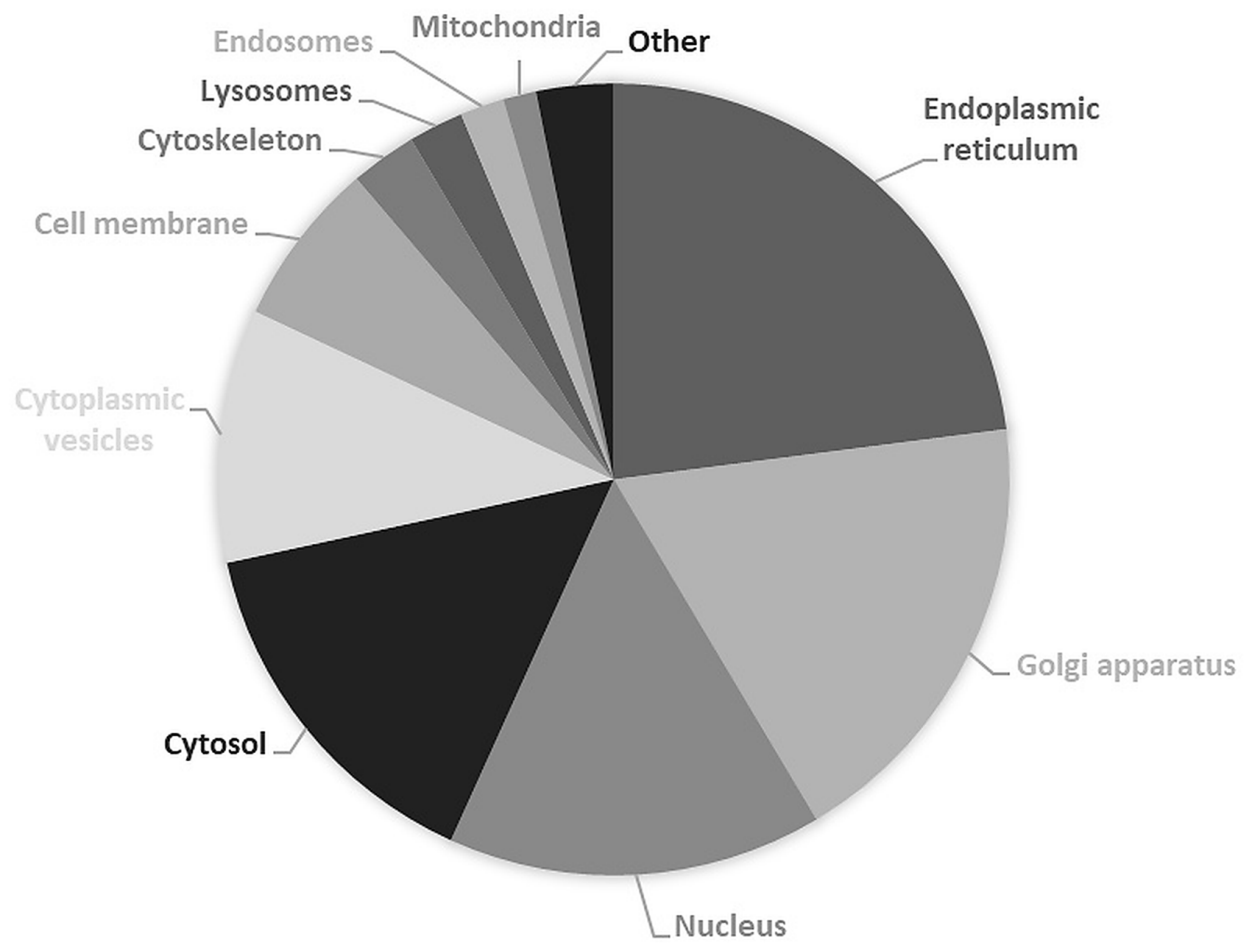
Subcellular localization of proteins encoded by causal genes of congenital disorders of glycosylation. Results are expressed in percentage of total expressed proteins. Data from UniProt/Swiss-Prot (UniProt Consortium, 2025). Explanation in the text.

**Figure 5:**
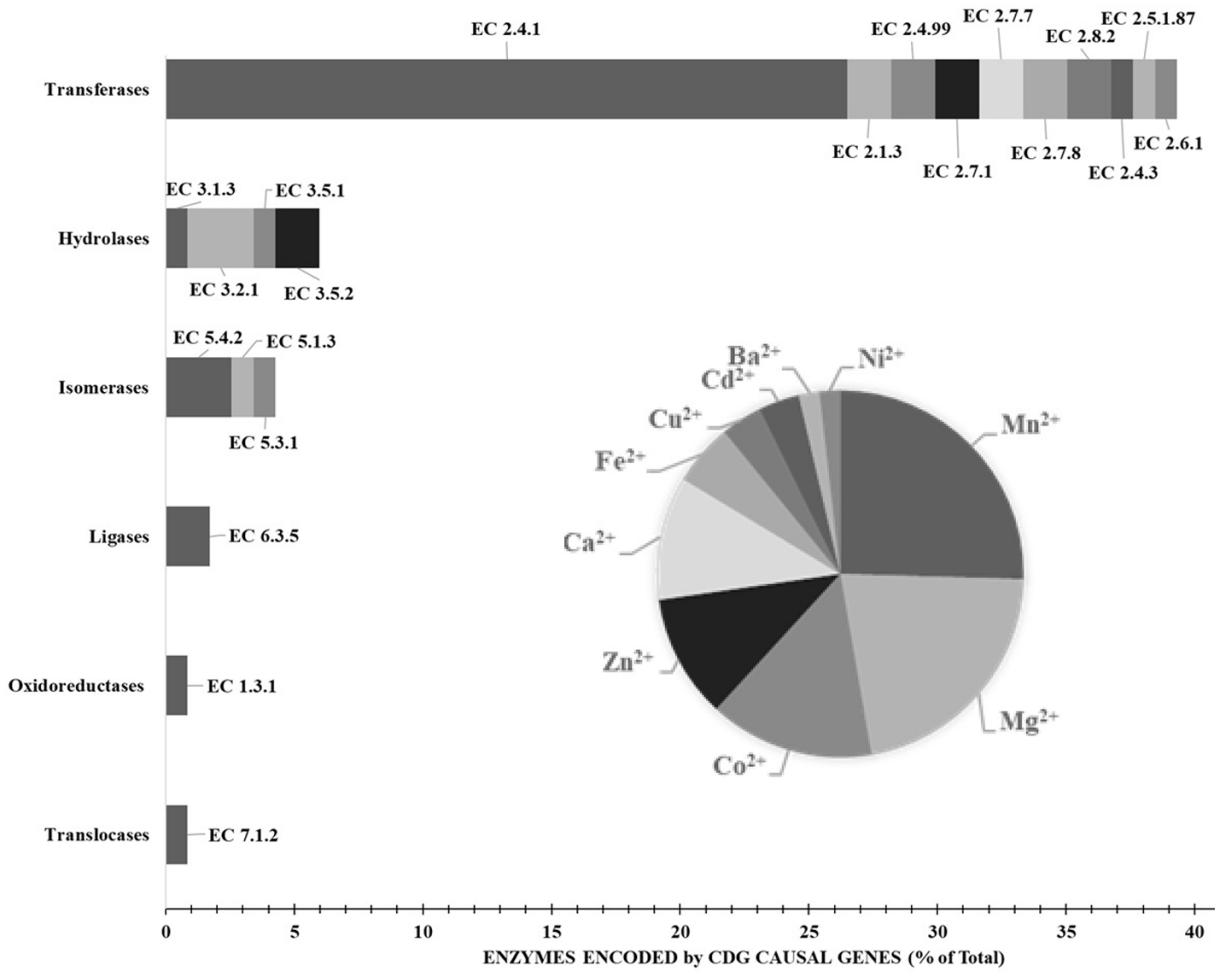
Enzymes encoded by causal genes of congenital disorders of glycosylation. The insert shows the divalent cation requirements of the enzymes. Results are expressed in percentage of total expressed enzymes. Data from BRENDA Enzyme (Schomburg et al., 2017) and Nomenclature Committee of IUBMB (RRID: SCR_003024). Explanation in the text.

It is therefore unsurprising that causal genes of CDG encode proteins whose subcellular distribution is mostly observed in endoplasmic reticulum (51%) and Golgi apparatus (41%), followed by nucleus (34%), cytosol (33%) and cytoplasmic vesicles (23%) (Figure 4). Likewise, approximately 85% of the proteins currently considered responsible for CDG are enzymes (Figure 5), with glycosyltransferases constituting the most abundant group (25%). Interestingly and no less important is the fact that several enzymes require divalent cations to exhibit maximum activity, for instance fucose kinase (EC 2.7.1.52), mannose phosphate isomerase (EC 5.3.1.8), protein *O*-fucosyltransferase 1 (EC 2.4.1.221), collagen β(1-*O*)galactosyltransferase 1 (EC 2.4.1.50), endoplasmic reticulum degradation enhancing α-mannosidase like protein 3 (EC 3.2.1.113), chondroitin sulfate synthase 1 (EC 2.4.1.175), β-1,3-glucuronyltransferase 3 (EC 2.4.1.135), mannosidase α class 2C member 1 (EC 3.2.1.24), 3′(2′),5′-bisphosphate nucleotidase 2 (EC 3.1.3.7), GDP-mannose pyrophosphorylase B (EC 2.7.7.13), phosphoglucomutase 1 (EC 5.4.2.2), glucosamine (UDP-*N*-acetyl)-2-epimerase/*N*-acetylmannosamine kinase (EC 2.7.1.60), β-1,4-galactosyltransferase 1 (EC 2.4.1.133), polypeptide *N*-acetylgalactosaminyltransferase 3 (EC 2.4.1.41), LFNG *O*-fucosylpeptide 3-β-*N*-acetylglucosaminyltransferase (EC 2.4.1.222), α-1,6-mannosyl-glycoprotein 2-β-*N*-acetylglucosaminyltransferase (EC 2.4.1.143), β − 1,3-galactosyltransferase 6 (EC 2.4.1.134), polypeptide *N*-acetylgalactosaminyltransferase 2 (EC 2.4.1.41) and phosphomannomutase 2 (EC:5.4.2.8). As is shown in the Figure 5 insert, Mn^2+^, especially, plays a crucial role in enzyme catalysis in the context of glycosylation. Interestingly, the enzymes *N*-glycanase 1 (EC 3.5.1.52) and α-mannosidase (EC3.2.1.24), which are involved in congenital disorders of deglycosylation (Sonoda et al., 2024) also depend on divalent cations, namely Zn^2+^ and Co^2+^.

**Figure 6:**
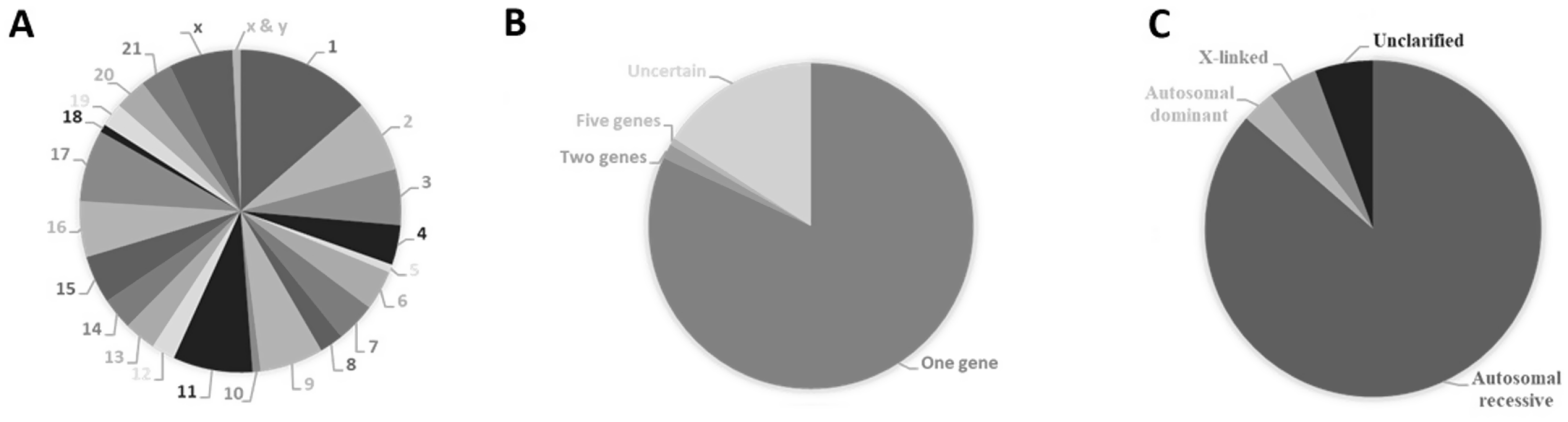
Causal genes of congenital disorders of glycosylation. **(A)** Percentual distribution of causal genes according to their cytogenetic location. **(B)** Percentage of congenital heart diseases caused by variations within a single gene (monogenic) or multiple genes (polygenic). **(C)** Types of Inheritance of congenital disorders of glycosylation expressed in percentage. Data from The Monarch Initiative database (Putman et al., 2024). Explanation in the text.

The known number of genes that cause disorder of glycosylation diseases is still constantly growing and can be found on almost all chromosomes but are not uniformly distributed. Chromosomes 1, 2, 11 and 17 appear to contain the most causal genes (Figure 6A). Like in the case of congenital heart diseases, CDG appear to be mostly monogenic (Figure 6B) but with autosomal recessive inheritance (Figure 6C).

Although not yet fully curated by ClinGen, The Congenital Disorders of Glycosylation Gene Curation Expert Panel has shown interest in TMEM165-CDG (MONDO:0013870). According to the Description of Monarch Initiative, this “*autosomal recessive disease is a form of congenital disorders of N-linked glycosylation characterized by a psychomotor delay-dysmorphism (pectus carinatum, dorsolumbar kyphosis and severe sinistroconvex scoliosis, short distal phalanges, genua* var*a, pedes planovalgi syndrome) with postnatal growth deficiency and major spondylo-, epi-, and metaphyseal skeletal involvement. Additional features include facial dysmorphism (midface hypoplasia, internal strabism of the right eye, low-set ears, moderately high arched palate, small teeth), nephrotic syndrome, cardiac defects, and feeding problems*.” Given the importance of ionic homeostasis for glycosylation and cardiomyocyte activity, this review will focus on the TMEM165 protein, highlighting its structure and function to emphasize the main properties in the context of cardiomyopathies associated with CDG.

## TMEM165 is a member of the uncharacterized protein family 0016 with undisputed functions

### Structural and functional motifs of the evolution conserved TMEM165

The human transmembrane protein 165 (HsTMEM165) gene (HGNC:30760, Gene ID: 55858, updated on 3-May-2025), also known as FT27, GDT1, CDG2K, TPARL, TMPT27 and SLC64A1, consists of six exons located on chromosome 4q12. This gene has at least 12 transcripts (splice variants) and 204 orthologues. HsTMEM165 was identified in 2012 through a combination of autozygosity mapping and expression analysis in two siblings with an abnormal serum-transferrin isoelectric focusing test (type 2) and a peculiar skeletal phenotype with epiphyseal, metaphyseal and diaphyseal dysplasia (Foulquier et al., 2012). The identification of this gene guided the establishment of the CaCA2 family (TC#2. A.106 Ca^2+^:H^+^ Antiporter-2 (CaCA2); SwissProt family Uncharacterized protein family UPF0016; Pfam PF01169; Prosite entry PDOC00934), a highly conserved protein family throughout evolution, with putative members identified in virtually all eukaryotes and in many bacteria (Demaegd et al., 2014). The SwissProt family UPF0016 contains hundreds of protein sequences, most of them are predicted proteins of unknow function. Phylogenetic studies assigned two highly conserved motifs E-*φ*-G-D-[KR]-[TS] as its consensus pattern. HsTMEM165 (TC#2. A.106.2.2 Ca^2+^/Mn^2+^/Mg^2+^: H^+^ antiporter, Q9HC07) and ScGDT1 from *Saccharomyces cerevisiae* are the most representative members of this protein family (Colinet et al., 2016; Colinet et al., 2017; Stribny et al., 2020; Xu et al., 2023). HsTMEM165 gene encodes a polypeptide chain protein that appears to contain six transmembrane helical segments (TMS 1: 90–110; TMS 2: 127–147; TMS 3: 152–172; TMS 4: 229–249; TMS 5: 268–288; TMS 6: 300–320), four lumenal domains (LD 1: 34–89; LD 2: 148–151; LD 3: 250–267; LD 4: 321–324) and three cytoplasmic domains (CD 1: 111–126; CD 2: 173–228; CD 3:289–299; Figure 7).

**Figure 7:**
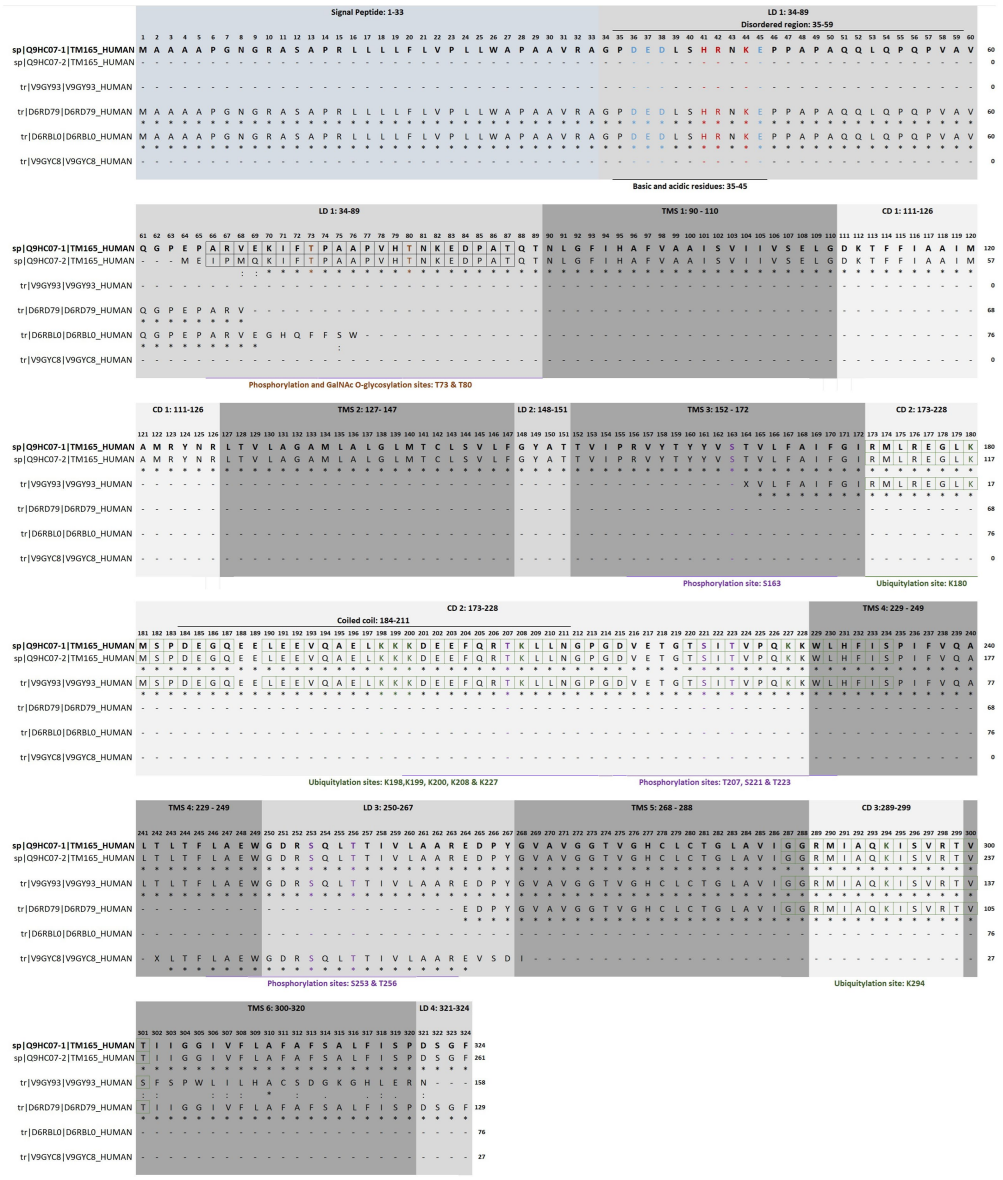
Topology, functional motifs and putative post-translational modifications of human TMEM165. Full sequence alignment of isoform 1 (sp|Q9HC07–1|TM165_HUMAN), isoform 2 (sp|Q9HC07–2|TM165_HUMAN) and computationally mapped potential isoforms (tr|V9GY93|V9GY93_HUMAN, tr|D6RD79|D6RD79_HUMAN, tr|D6RBL0|D6RBL0_HUMAN and tr|V9GYC8|V9GYC8_HUMAN) using Clustal Omega. LD, lumenal domain; CD, cytoplasmic domain; TMS, transmembrane helical segments. The residues that are strictly conserved are indicated with an asterisk below the alignment, whereas the semi-conserved and conserved are indicated with one or two dots, respectively. The amino acids highlighted in red and blue, correspond to basic and acidic residues, respectively. Putative sites of glycosylation, phosphorylation and ubiquitylation are highlighted in brown, purple and green, respectively. The boxes indicate the respective recognition sequence motifs. Data from UniProt/Swiss-Prot (UniProt Consortium, 2025) and PhosphoSitePlus® v6.7.9 y (Hornbeck et al., 2015). Explanation in the text.

As previously mentioned, the highly conserved motif E-φ-G-D-[KR]-[TS] is the consensus pattern of the SwissProt family, that is usually flanked by two hydrophobic regions containing 16–20 residues on the N-terminal side and 8–9 residues on the C-terminal side and also comprise additional hydrophobic regions of approximately 25–28 and 16–19 residues (Foulquier et al., 2012). Probably, in isoform 1 the motif 1 (108ELGDKT113) faces the cytosol, while the motif 2 (248EWGDRS253) is exposed to the organelle luminal side. Motif 2 is predicted to be involved in the transport function (Colinet et al., 2017). An extensive study by sitedirected mutagenesis of Gdt1p (the budding yeast representative of CaCA2) revealed that the conserved residues of the acidic cytosolic loop and of the predicted central transmembrane domains are important for calcium tolerance and transport activity (Demaegd et al., 2014; Colinet et al., 2017; Lebredonchel et al., 2019b). While the exact functions of the two conserved motifs remain elusive (Legrand et al., 2023), it was suggested that the acidic amino acids in the motif facing the cytosol play a role in the Mn^2+^ sensitivity of TMEM165 (Dulary et al., 2018; Lebredonchel et al., 2019b). Moreover, the aspartic residues of the two conserved motifs, likely constitute the cation binding sites of TMEM165, and hence play a crucial role in Ca^2+^ and Mn^2+^ transport (Dulary et al., 2017). These structural and functional features closely resemble those of the Ca^2+^:cation antiporter (CaCA) family (TC #2.19), which is composed of K^+^-independent Na^+^/Ca^2+^ exchangers (NCXs), cation/Ca^2+^ exchangers (CCXs), YbrG transporters and cation exchangers (CAXs) (Nicoll et al., 1996; Schwarz and Benzer, 1997; Hirschi, 2001; Winkfein et al., 2003; Cai and Lytton, 2004; Kamiya and Maeshima, 2004; Wu et al., 2013; Singh et al., 2015).

### Post-translational modifications of TMEM165 and regulatory features of its isoforms

The initial 33 amino acid residues of the polypeptide chain are thought to be the cleavable secretory pathway signal peptide (Signal Peptide 1–33), and so the mature forms of the protein are shorter. The isoform 1 (Q9HC07–1) corresponds to the canonical sequence, and isoform 2 (Q9HC07–2) is a polypeptide chain shortened by 63 amino acid residues from the N-terminal region (Ota et al., 2004). Therefore, the signal peptide and the first disordered region (Pro35-Ala59) are missing in isoform 2. Indeed, it is in the N-terminal region that members of the UPF0016 protein family exhibit the highest variability (Demaegd et al., 2014; Schneider et al., 2016). This region enriched in acidic (Asp36; Glu37, Asp38 and Glu45) and basic (His41, Arg42 and Lys44) residues is a putative autoinhibitory/regulatory domain by similarity to the consensus sequence found in some members of other Ca^2+^/H^+^ antiporter families (Pittman and Hirschi, 2001). This feature may be of great interest. The binding of calmodulin, a multifaceted Ca^2+^-binding regulatory protein, is expected to cause activation. Conversely phosphorylation at the neighbor Thr73 and/or Thr80 by either calcium- or calcium/calmodulin-dependent protein kinases should have an inhibitory action. This makes TMEM165 responsive to changes in Ca^2+^ concentrations. Ca^2+^/calmodulin-dependent protein kinase IIα pathway is activated in TMEM165-knockout mouse ATDC5 cells (Khan et al., 2021). The same threonine residues are also possible GalNAc *O*-glycosylation sites, if we consider the consensus ARVEKIFtPAAPVHt and tPAAPVHtNKEDPAT, respectively (Steentoft et al., 2013).

It is conceivable that HsTMEM165 isoform 1 could also be regulated through competition between glycosylation and phosphorylation events, whereas the isoform 2, produced by alternative splicing, could not. Analysis of the PhosphoSitePlus database showed several potentially phosphorylatable residues along the sequence (Figure 7; Table 2). Some amino acid residues (such as Thr207) located in or near the coiled-coil domain Asp184-Arg211 deserve attention, since these structures also function as protein–protein interaction motifs. Most likely, isoform 1 also contains a disordered region (Pro35-Ala59) suitable for protein interaction. In addition to phosphorylation and glycosylation, the analysis also showed ubiquitylation sites (Figure 7; Table 2). Ubiquitylation is a well-studied post-translational modification, that plays versatile roles in protein functions ranging from protein degradation, protein–protein interaction to subcellular localization. The coiled-coil domain is also enriched in predicted ubiquitylation sites (Lys180, Lys198, Lys199, Lys200, Lys208, Lys227 and Lys294).

**Table 2:** Putative post-translational modifications of human TMEM165.

Position	Recognition sequence motif	References
Phosphorylation and glycosylation sites		
T73-ga	ARVEKIFtPAAPVH ARVEKIFtPAAPVHt ARVEKIFtPAAPVHt	Steentoft et al. (2013)
T80-ga	tPAAPVHtNKEDPAT	Steentoft et al. (2013)
Phosphorylation sites		
S163	RVYTYYVsTVLFAIF	Hornbeck et al. (2015)
T207	kDEEFQRtkLLNGPG	Hornbeck et al. (2012)
S221	GDVETGTsITVPQKK	Nowak et al. (2015); Ochoa et al. (2020)
T223	VETGTSItVPQKKWL	Nowak et al. (2015); Ochoa et al. (2020)
S253	LAEWGDRsQLtTIVL	Hornbeck et al. (2012); Alcolea et al. (2012)
T256	WGDRsQLtTIVLAAR	Hornbeck et al. (2012)
Ubiquitylation sites		
K180	RMLREGLkMSPDEGQ	Kim et al. (2011)
K198	EEVQAELkkkDEEFQ	Kim et al. (2011); Akimov et al. (2018)
K199	EVQAELkkkDEEFQR	Akimov et al. (2018)
K200	VQAELkkkDEEFQRt	
K208	DEEFQRtkLLNGPGD	Povlsen et al. (2012); Kim et al. (2011); Udeshi et al. (2013); Akimov et al. (2018)
K227	TSITVPQkKWLHFIS	Kim et al. (2011); Akimov et al. (2018)
K294	GGRMIAQkISVRTVT	Wagner et al. (2011)
Total	15	

Source: PhosphoSitePlus® v6.7.9 y (Hornbeck et al., 2015).

Whether TMEM165 isoforms may exhibit distinct regulatory mechanisms when interacting with other proteins at the N-terminal region remains an open question (Demaegd et al., 2013). This is a common but distinctive feature among members of Ca^2+^/H^+^ antiporter families. In fact, regulatory mechanisms of CAX members involve activation by dimerization and interaction with small proteins, CAX interacting proteins (CXIPs). In response to a specific stimulus, CXIPs bind to the Nterminal CAX domain triggering the release of autoinhibition and activation of cation transport (Pittman and Hirschi, 2024). There are four computationally mapped potential HsTMEM165 isoform sequences, namely V9GY93 158 a.a., D6RD79 (129 a.a.) D6RBL0 (76 a.a.) and V9GYC8 (27 a.a.). As shown in Figure 7, these predicted isoforms are likely nonfunctional, because they either lack transmembrane segments (V9GYC8 and D6RBL0) or family consensus patterns (D6RD79). Like isoform 2 (Q9HC07–2), the transcript described in hypothalamus (BI457666) that corresponds to the single-pass membrane isoform D6RD79, only contains one family consensus pattern (248EWGDRS253). The existence of more than one TMEM165 splice transcript isoform was demonstrated experimentally (Krzewinski-Recchi et al., 2017). The 129 a.a. (D6RD79) and 259 a.a. (Q9HC07–2) protein isoforms are localized in the endoplasmic reticulum, as shown by colocalization assays using two endoplasmic reticulum markers (calnexin and glucosidase II) in human brain tissues. Interestingly, the 129 a.a. protein can form homodimers, by the formation of disulfide bond between cysteines, and is likely expressed at low levels in all human cells and tissues but predominantly in human brain. The expression of the 259 a.a. protein appears to be restricted to the temporal lobe of the human brain (Krzewinski-Recchi et al., 2017). In the same set of experiments, the isoform 1 was shown to be localized in the Golgi apparatus. It was hypothesized that the 129 a.a. protein could also participate in the regulation of ionic homeostasis in the endoplasmic reticulum, because of its subcellular ubiquitous expression. Thereby, TMEM165 splice transcripts could participate in the fine regulation of TMEM165 isoforms’ functions and subcellular distribution in a very restrictive and tissue specific way (Krzewinski-Recchi et al., 2017).

### Interactome and trafficking partners of TMEM165

**Figure 8:**
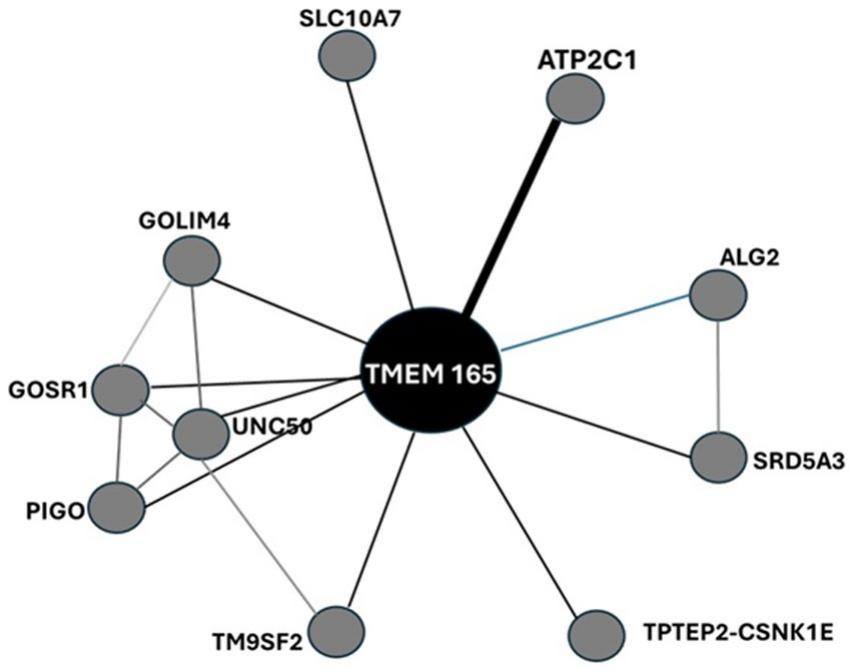
Protein–protein interaction network of HsTMEM165 visualized by STRING. The nodes indicate proteins and edges indicate the number of interactions. The interaction type, combined confidence and co-expression score are provided in Supplementary Table 3S. Data from STRING, ComPPI and TFLink databases (Veres et al., 2015; Szklarczyk et al., 2021; Liska et al., 2022). Explanation in the text.

Protein interactions across tissues, cellular subpopulations and subcellular compartments form specific subnetworks that are tightly regulated in an organ/tissue-specific manner and provide insights into physiological and pathophysiological functions of proteins. Interactions of HsTMEM165 with putative structural/functional partners are anticipated by evaluating its interactome generated by STRING analysis, as well as ComPPI and TFlink (Figure 8; Supplementary Table S3).

Eventually, TRIM25 and CUL3 could be engaged in ubiquitination of TMEM165 and its subsequent proteasomal degradation. Given that TRIM25 functions as a ubiquitin E3 ligase and Cullin3-based ubiquitin E3 ligase complexes catalyze the transfer of ubiquitin to target substrate proteins.

The mitogen-activated protein kinases (MAPKs) are among the most widespread cellular regulators that mediate responses to a variety of stimuli, whose signaling fidelity requires proper *N*-glycosylation (Lien et al., 2013). HsTMEM165 disfunction leads to severe *N*-glycosylation defects. TMEM165 appears also to be linked with MAPK signaling pathways during cytotoxic events through functional interaction with the Golgi SNAP receptor complex member 1, a soluble NSF (*N*-ethylmaleimide-sensitive factor) attachment protein receptor (GOSR1). GOSR1 participates in docking and fusion stage of endoplasmic reticulum to *cis*-Golgi transport of proteins. The Golgi integral membrane protein 4 (GOLIM4) is a multispanning membrane protein of the postsynaptic Golgi apparatus region and other intracellular compartments. It also plays a role in protein trafficking by mediating protein transport along the late endosome-bypass pathway from the early endosome to the Golgi apparatus. The transmembrane 9 superfamily member 2 (TM9SF2) is involved in the transport of small molecules and in the regulation of heparan sulfate proteoglycan biosynthesis (Schimmöller et al., 1998). Similarly, Sodium/bile acid cotransporter 7 (SLC10A7), another multi-pass membrane protein of endoplasmic reticulum and Golgi apparatus, also found in the plasma membrane, is essential in the regulation of Ca^2+^ homeostasis and in the trafficking and biosynthesis of glycosaminoglycans and glycoproteins, particularly to covalent attachment of heparin chains via *O*-linkage. GPI ethanolamine phosphate transferase 3 (PIGO), polyprenol reductase (SRD5A3) and *α*-1,3/1,6-mannosyltransferase (ALG2) are enzymes. The former is a key enzyme in the formation of a glycosylphosphatidylinositol (GPI) anchor that attaches proteins to biomembranes. SRD5A3 plays a key role in early steps of protein *N*-linked glycosylation, since it converts polyprenol into dolichol, used for the synthesis of oligosaccharide precursors for N-glycosylation. On the other hand, ALG2 mannosylates Man(2)GlcNAc(2)-dolichol diphosphate and Man(1)GlcNAc(2)-dolichol diphosphate to form Man(3)GlcNAc(2)-dolichol diphosphate.

Calcium-transporting ATPase type 2C member 1 (ATP2C1) belongs to the P-type Ca^2+^ transporter family that undergoes covalent phosphorylation during the Post-Albers reaction cycle (EC 7.2.2.10). Human Ca^2+^ pumps can be split into three main types that are found in the plasma membrane (PMCAs), the endoplasmic/sarcoplasmic reticulum (SERCAs) and along the secretory pathway (SPCAs). SPCAs are encoded by HsATP2C1 and HsATP2C2 genes, while there are three major SERCA paralogs (SERCA1-3), which are expressed at various levels in different cell types. In contrast to SPCAs that mediate ATP-powered uphill transport of either Ca^2+^ or Mn^2+^ from cytosol into the lumen of Golgi apparatus, SERCAs exhibit high Ca^2+^ selectivity (Dode et al., 2005). The endogenous interaction between TMEM165 and the Golgi P type Ca^2+^/Mn^2+^ -ATPase (SPCA1), or at least their close intracellular localization, was shown in Hap1 cells by proximity ligation assays (PLA) (Lebredonchel et al., 2019a). At least, these two membrane proteins seem functionally linked, since TMEM165 is constitutively degraded in lysosomes when SPCA1A is absent (Lebredonchel et al., 2019a). Colocalization experiments with the Golgi marker GM130 confirmed the subcellular Golgi localization of HsTMEM165 and its colocalization with SPCA1 in human fibroblasts and keratinocyte (Roy et al., 2020). These results are in line with the involvement of Ca^2+^-ATPases in the rescue of TMEM165 subcellular location and in the Golgi *N*-glycosylation defects in TMEM165 KO cells by extracellular Mn^2+^ (Houdou et al., 2019). Certainly, the interaction of TMEM165 and SPCA /SERCA pumps impacts on the regulation of intracellular Ca^2+^/Mn^2+^ homeostasis, which is crucial for many fundamental cellular processes such as muscle contraction, protein secretion and glycosylation (Roy et al., 2020).

Analysis of a TMEM165 structural model built using AlphaFold 2 suggested that it can interact with heterotetrameric adaptor protein (AP) complexes (AP1, AP2, AP3 or AP4), which recruit clathrin to initiate the formation of coated vesicles, consistent with dynamic cycling within the secretory/endo-lysosomal system (Legrand et al., 2023). Apart from the presence of the 124YNRL127 sequence in HsTMEM165 polypeptide chain, a tyrosine-based lysosomal-targeting signal YXXØ, there is no other evidence supporting this interesting hypothesis. However, TMEM165 activity. These are two words. as a cytosolic Ca^2+^ regulator should impact AP complexes, since Ca^2+^/calmodulin acts as a protein linker that regulates sorting cargo proteins into transport vesicles for intracellular trafficking by AP complexes.

### Subcellular location and tissue expression of TMEM165

HsTMEM165 is considered to be the unique Golgi-localized Ca^2+^/H^+^ antiporter in human cells and appears to be predominantly a Golgi-resident protein (Demaegd et al., 2013). Immunocytochemistry using Golgi marker antibodies is often the method selected to quantify the distribution of TMEM165 in cells and tissues. TMEM165 was found at the plasma membrane (Foulquier et al., 2012), and notable fractions reside in endoplasmic reticulum, perinucleus, lysosomes, endosomes, secretory vesicles and virtually in all acidic cytoplasmic vesicular compartments (Foulquier et al., 2012; Dulary et al., 2017; Potelle et al., 2017; Khan et al., 2021; Zajac et al., 2024).

**Figure 9:**
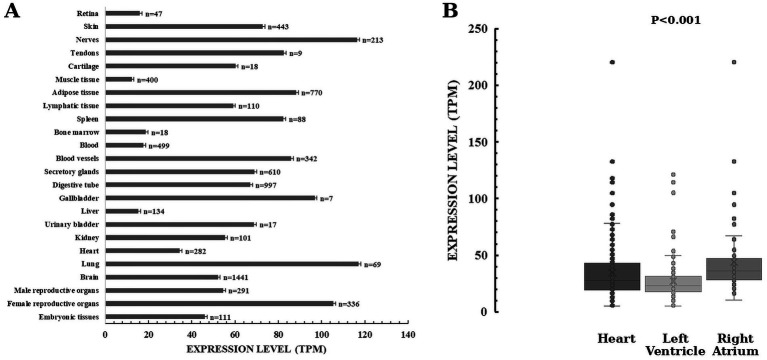
Expression profiles of TMEM165 in human body. HsTMEM165 expression levels in organs and tissues **(A)** and in heart regions **(B)**. Numbers denote the total number of analyzed samples. Data from Genotype-Tissue Expression (GTEx) Common Fund Project and The Human Protein Atlas (Digre and Lindskog, 2023). Explanation in the text.

As it can be seen in Figure 9A, TMEM165 is ubiquitous in human organs and tissues from the embryonic stage to adulthood (Schulte Althoff et al., 2016). Most likely, the quantification of TMEM165 expression using RNA-seq is underestimated due to the still incipient knowledge of isoform diversity and technical limitations. The powerful RNA-seq has limitations in dealing with short transcripts and alternative splicing. In the Human Protein Atlas tau specificity score of HsTMEM165 is 0.36, indicating low specificity of its expression across cells and tissues. HsTMEM165 exhibits relative high expression levels in lungs and nerves, which is in concordance with the identified *oligodendrocyte enriched brain cluster and unknown function respiratory epithelial cells cluster*, respectively. In the *oligodendrocyte-enriched brain cluster* oligodendrocytes and their precursor cells are the predominant cell populations. These regions of the central nervous system are characterized by a high concentration of oligodendrocytes, myelinated axons and myelin-forming cells. In response to neuronal activity, changes in myelination within these regions can influence the speed and synchrony of neural signal transmission, thereby affecting motor function, learning, and memory. TMEM165 deficiency cause neurological alterations in patients with TMEM165-CDG (Table 3). Reported TMEM165-CDG cases frequently include global developmental/psychomotor delay (e.g., psychomotor delay/hypotonia, seizures) and CNS abnormalities (e.g., white-matter changes). In matter of fact, TMEM165 deficiency correlate with neurological alterations, and severe psychomotor retardation is among prominent clinical findings. In individual patient histories with confirmed diagnosis of TMEM165-CDG preeminent neurological findings include neurodevelopmental and severe psychomotor delays, psychomotor-dysmorphism syndrome, neuromotor regression, enlarged cerebral ventricles, cerebellar atrophy, white-matter abnormalities, pituitary hypoplasia, mild facial dysmorphism and nystagmus (Foulquier et al., 2012; Zeevaert et al., 2013; Schulte Althoff et al., 2016; Kılavuz et al., 2023; Durin et al., 2024).

**Table 3:** Clinical cases of patients with a confirmed diagnosis of TMEM165-CDG with neurological findings (Illustrative individual patient histories).

Patients		Neurological alterations	Other clinical features	References
Two siblings	Adolescence diagnosed at the age of 19 years.	Severe psychomotor delay; Psychomotor retardation (independently walking at 2 years of age); White-matter abnormalities; Pituitary hypoplasia; Acquired microcephaly.	Growth retardation; Partial growth-hormone deficiency; Feeding problems/ pseudo-obstruction; Fat excess; Failure to thrive; Skeletal dysplasia; Severe dysmorphy; Hypotonia; Eye abnormalities; Hepatosplenomegaly; Osteoporosis; Skeletal dysplasia; Joint laxity; Midface hypoplasia.	Foulquier et al. (2012)
	Death occurred at 14 months of age.	Severe psychomotor delay; Acquired microcephaly; EEG abnormal.	Growth retardation; Partial growth-hormone deficiency; Severe dysmorphy; Hypotonia; Eye abnormalities; Hepatosplenomegaly; Feeding problems/ pseudo-obstruction; Fat excess; Failure to thrive; Osteoporosis; Skeletal dysplasia; Joint laxity; Midface hypoplasia; Thrombopenia; Unexplained fever episodes; Acute infectious shock.	
Child diagnosed at the age of 4 years.		Severe psychomotor delay; Acquired microcephaly; Convulsions; Transient epilepsy.	Growth retardation; Feeding problems / pseudo-obstruction; Failure to thrive; Severe dwarfism; Dysmorphy; Hypotonia; Hepatomegaly; Skeletal dysplasia; Thrombopenia; Unexplained restrictive lung pathology (artificial ventilation via tracheostomy); Unexplained fever episodes.	
Child diagnosed at the age of 11 years.		Severe psychomotor delay (4-year-old’s psychomotor level at the age of 9 years); Acquired microcephaly.	Growth retardation; Failure to thrive; Mild rhizomelia; Dysmorphy; Hypotonia; Hepatomegaly; Haemolytic uremic syndrome; Thrombopenia.	
Two siblings	Child diagnosed at the age of 11 years.	Psychomotor-dysmorphism syndrome; Psychomotor retardation (unsupported sitting at 9 months, independent walking at 2 years, and first words at 18 months); Brain atrophy; Enlarged ventricles; Periventricular and subcortical white matter abnormalities; Hyporeflexia; Relative hypoplasia of the anterior pituitary, and absence of the normal hyperintensity of the posterior pituitary.	Postnatal growth deficiency; Partial growth hormone deficiency; Dysmorphim; Major spondylo-, epi-, and metaphyseal skeletal involvement; Muscular hypotrophy; Joint hyperlaxity; Waddling gait; Hepatosplenomegaly; Fat excess; Feeding problems; Right convergent strabism and “in fundo” temporal epithelial pigment alterations.	Zeevaert et al. (2013)
	Infant diagnosed at the age of 5 months	Psychomotor-dysmorphism syndrome; Psychomotor delay (unsupported sit for a few moments and roll over from the side to the prone or supine position at 11 months); Decreased amplitude of motor nerve conduction with sequelae of past denervation; Hyporeflexia; Large fontanel; Ptosis of the right upper eyelid.	Postnatal growth deficiency; Partial growth hormone deficiency; Dysmorphim; Hypotonia; Generalized osteoporosis; Joint hyperlaxity; Discrete irregular metaphyses of the long bones; Discrete plathyspondyly; Hip dysplasia; Broad iliac wings; Horizontal acetabular roofs; Subluxation of the right femur; Torticollis toward the left; Rather large lowly implanted ears, Long philtrum; High arched palate; Short and broad neck; Broad thorax with increased nipple distance; Right hip dysplasia; Sacral dimple; Absence of the second-toe nails; External strabism of the left eye; Macular epithelial pigment alteration; Recurrent, unexplained fever up to 40 °C during 4 months; Hepatosplenomegaly; Splenomegaly; Pyelonephritis; Obesity; Slightly enlarged abdomen; Vomiting; Infection-related phase of respiratory insufficiency; Enterocolitis-related episode of shock.	
Infant diagnosed at the age of 3 months		Psychomotor-dysmorphism syndrome; Psychomotor delay (at age of 6 years does not follow normally with his eyes, does not grasp for objects, and cannot sit unsupported); Epilepsy; Irritability with opisthotonus; Macrocephaly; J-like sella turcica; Invisible neural pituitary gland; De- or dysmyelination; Brisk tendon reflexes.	Postnatal growth deficiency; Partial growth hormone deficiency; Dysmorphim; Small open ductus Botalli; Small patent foramen ovale; Small pericardial effusion; Blepharitis of the right eye; Hepatomegaly; Feeding difficulties; Diarrhea; Distended, tympanic abdomen; Craniofacial dysmorphism (relative, tongue protrusion, anti-mongoloid slanting of the eyes, flat nose, and relatively large and posteriorly rotated ears); Restrictive lung pathology (long-term invasive ventilation via tracheostomy); Body adiposity; Hypotonia; Muscular weakness; Generalized osteopenia; Hypoplasia of the skull base; Mild anterior breaking of vertebrae (D11, D12, L1, and L2); Broad radial and ulnar metaphyses; Strongly underdeveloped carpal bones, plump and broad phalanges; Horizontal acetabula; Very discrete opacification of the right proximal femur epiphysis; No ossification of the left proximal femur epiphysis; Broad proximal femur metaphyses; Strongly underdeveloped distal femoral and proximal tibial epiphyses; Joint hyperlaxity (with cracking noise on mobilization); Regularly bouts of fever up to 38.5 °C without evidence for infection.	
Six-month-old fetus (prenatal diagnostics).		Enlarged lateral and third ventricles; Large, temporarily tensed fontanel; Muscular hypertonia with opisthotonic posture; Sundown position of the eyes; Facial dysmorphia.	Weak abdominal wall; Feeding problems; Cardiac defects (small apical ventricular septal defect, patent foramen ovale patent ductus arteriosus with mild signs of right ventricular hypertrophy); Mild proteinuria nephrotic syndrome / nephrotic syndrome / slowly progressive renal failure.	Schulte Althoff et al. (2016)
Three Siblings	Child diagnosed at the age of 3 years.	Neurodevelopmental delay (unable to speak); Psychomotor retardation / Neuromotor Regression; Cerebellar Atrophy; Facial dysmorphia.	Hypotonia; Polyuria; Polydipsia; Electrolyte imbalance; Bone dysplasias; Prominent renal parenchymal disease; Pneumonia (Candida spp.); Hepatomegaly; Thrombocytopenia; Coagulation disorders.	Kılavuz et al. (2023)
	Child diagnosed at the age of 8 years.	Neurodevelopmental delay (unable to speak); Psychomotor retardation / Neuromotor Regression; Cerebellar Atrophy / Cerebellar hypoplasia / Subarachnoid space widening; Facial dysmorphia.	Meconium aspiration syndrome; Growth retardation; Bone dysplasias; Pes equinovarus (walk with support); Prominent renal parenchymal disease; Hepatomegaly; Thrombocytopenia.	
	Infant diagnosed at the age of 11 months.	Neurodevelopmental delay; Psychomotor retardation / Neuromotor Regression; Cerebellar Atrophy; Facial dysmorphia.	Hypotonia; Vomiting; Electrolyte imbalance; Hepatomegaly; Bone dysplasias; Thrombocytopenia; Prominent renal parenchymal disease; Pyelonephritis attack (Candida spp.); Chronic renal failure.	
Infant diagnosed at the age of 3 months.		Slight developmental delays; Discreet delay in psychomotor development; Mild facial dysmorphism; Nystagmus with a good eye tracking.	Digestive symptoms; Hemostasis defect; Retinoschisis; Bone impairment.	Durin et al. (2024)

In general, cell pools of undifferentiated mitotic cells in the same tissue appear to express more TMEM165 than do their differentiated counterparts. For example, in heart muscle, the enrichment of HsTMEM165 expression in undifferentiated cells is 1.5 times higher than in cardiomyocytes. Therefore, it is reasonable to emphasize the importance of TMEM165 during embryonic development, and tissue renewal, regeneration and repair. Besides causing CDG, HsTMEM165 is functionally involved in several other pathologies, including cancer and mental health disorders (Dulary et al., 2017; Jankauskas et al., 2024). More intriguing is the difference in expression levels between regions of the same organ, unless TMEM165 activity. These are two words. has a very specific functional impact. Figure 9B shows that the expression of TMEM165 in the heart right atrium is higher than in the left ventricle. Whether TMEM165 expression is altered in diseased cardiac tissue is an open question. To date, there is no reproducible evidence that TMEM165 mRNA or protein is differentially expressed in human cardiovascular disease when compared to healthy individuals (Alimadadi et al., 2020). There are clear differences for the circular RNA derived from TMEM165 (circTMEM165), that promotes endothelial repair, implying regulatory roles beyond the protein coding gene. For instance, circTMEM165 is downregulated in serum and vascular tissues of patients with in-stent restenosis and atherosclerosis (Liu et al., 2024). Conversely, circTMEM165 is upregulated in peripheral blood of patients with myocardial infarction (Li et al., 2022).

### Physiological importance of TMEM165 transport functions

Over the last few decades an extraordinary similarity for proteins that convey the same function across the evolution was demonstrated. Patch-clamp analyses on human cells indicated that HsTMEM165 mediates Ca^2+^ transport, and defects in HsTMEM165 affected both pH and Ca^2+^ homeostasis (Demaegd et al., 2013). Electrophysiological patch-clamp recordings performed on human cells (HeLa cells stably overexpressing a RFP-tagged version of TMEM165) provided irrefutable evidence that TMEM165 mediates a tetraethylammonium insensitive, EGTA sensitive current, which is most likely linked to Ca^2+^ transport. Additionally, HsTMEM165 mediated acidification of intracellular acidic compartments was experimentally demonstrated by staining with an acidotropic probe using siRNA-treated HeLa cells and fibroblasts from different TMEM165-deficient patients (Demaegd et al., 2013). Using two-ion mapping and electrophysiology, it was demonstrated that TMEM165 imports Ca^2+^ into lysosomes in a pH dependent manner (Zajac et al., 2024). Direct biochemical evidence of TMEM165 mediated Ca^2+^ and Mn^2+^ influxes, that alter proper protein glycosylation, were obtained in *Saccharomyces cerevisiae* and in *Lactococcus lactis* expressing HsTMEM165 (Boukadida et al., 2014; Stribny et al., 2020). Ca^2+^ and Mn^2+^ transport capacity of HsTMEM165 orthologs were also clearly demonstrated by using fluorescent probes (Colinet et al., 2016; Thines et al., 2018; Stribny et al., 2020; Zajac et al., 2024).

Experimental evidence of the importance of TMEM165 in the context of Ca^2+^ and Mn^2+^ detoxification pathways to prevent toxic accumulation in the cytosol were obtained in the yeast and bacterium CDG models (Thines et al., 2018; Stribny et al., 2020). Once taken up by TMEM165, these ions can be directed out of the cell through secretory pathways (Potelle et al., 2017; Stribny et al., 2020). In rapid cholinergic synapses that operate at high frequency, following a brief period of stimulation, Ca^2+^ transiently accumulates in synaptic vesicles and is subsequently cleared from the terminal, most probably by exocytosis (Dunant et al., 2009). In a sophisticated study, Reinhardt and collaborators (Reinhardt et al., 2014) demonstrated the role of TMEM165 in milk Ca^2+^ secretion. Ca^2+^ in milk is transported across the apical membrane of mammary cells by PMCA2, but the remaining 40% milk Ca^2+^ arrive via the secretory pathway. Outstanding is the fact that TMEM165 expression was 25 times greater at the lactation peak than that of early pregnancy. Forced cessation of lactation resulted in a rapid 50% decline in TMEM165 expression at 24 h of involution and TMEM165 expression declined 95% at 96 h involution. Brain synaptic vesicles accumulate Ca^2+^ in two main manners, depending on the prevailing pCa and pH at the vicinity of the synaptic vesicle membrane (Gonçalves et al., 1998): by a P-type Ca^2+^-ATPase that works by pumping Ca^2+^ into the vesicles with high affinity for Ca^2+^ at neutral pH values, and by a Ca^2+^/ H^+^ antiport energized by the V-type H^+^-ATPase, which is largely operative at high Ca^2+^ concentrations. The cation selectivity of the synaptic vesicle Ca^2+^/H^+^ antiport is essentially determined by the size of the dehydrated cation that is transported into the vesicles (Gonçalves et al., 1999a). The Ca^2+^/H^+^ antiport is maximally active at basic pH values and exhibits a low affinity for Ca^2+^ that depends, essentially, on the ΔpH component of the electrochemical proton gradient (Gonçalves et al., 1999b). Although both pathways coexist, they would be rendered not simultaneously operative by their Ca^2+^ sensitivity. It is important to note that Ca^2+^/H^+^ antiport activation impacts neurotransmitter storage in secretory vesicles (Gonçalves et al., 2001). In excitable cells, neurotransmitter concentrative transport by vesicular transporters depends on vesicular lumen acidification. For instance, while transporters of GABA, glycine, glutamate and aspartate utilize either the ion concentration gradients or the electrical potential as driven forces, the transporters of acetylcholine and monoamines are exclusively proton concentration gradient dependent. Thereby, in contrast to glutamate and GABA, dopamine uptake is strongly inhibited by high Ca^2+^ concentrations.

### Energetic coupling of TMEM165 with proton pumps in secretory pathways

Secretory systems are an energy-rich store that drives membrane fusion. The V-type H^+^-ATPase (proton pump) is the energy interconverter in pH dependent membrane traffic processes, biogenesis of secretory vesicles and stimulus-secretion coupling paradigm, since the microenvironment at the secretion sites is likely dominated by proton-dependent bioenergetics. To assure the secondary active transport of Ca^2+^ into organelles with acidic lumenal pH, Ca^2+^/H^+^ antiporter (TMEM165) and a proton pump must be coexpressed at the same membranous compartment. Distinct isoforms of the subunit a of V-type H^+^-ATPase are detected in *trans* Golgi, secretory granules and plasma membrane (a1), *cis* Golgi and early endosome (a2), lysosome, secretory granules and plasma membrane (a3), and plasma membrane (a4) (Marshansky and Futai, 2008; Sun-Wada et al., 2004). The characteristics of Ca^2+^/H^+^ antiport resemble those of calcium transport into vacuole of yeast and plant cells (Demaegd et al., 2014), as well as into acidocalcisomes, the major intracellular calcium store in *Trypanosoma cruzi* and other parasites (Docampo and Moreno, 2011). Interestingly, the polypeptide composition and quaternary structure of the V-type ATPase (that energizes the Ca^2+^/H^+^ antiport) reveal remarkable phylogenetic conservation. The coexistence in the same cell membrane of Cation-ATPases and Cation(Ca)/H antiporters powered by a V-type H^+^-ATPase is a common observation, which probably constitutes the basic functional unit of divalent cation transport by all organelles with acidic luminal pH. Consistent with its interaction with SPCA1 described above, a remarkable match between the intracellular distribution of TMEM165 and SPCA1, a Ca^2+^-ATPase of the secretory pathway, has been reported in mouse mammary tissue (Reinhardt et al., 2014).

Glycosylation is also affected by physiological oscillations of intracellular Ca^2+^ concentrations, since Ca^2+^ is essential for proper vesicular trafficking through its role in membrane fusion, and for activity and stability of enzymes involved in glycosylation (Potelle et al., 2017; Dulary et al., 2018). Ca^2+^ also plays a main role in many signaling pathways. Interestingly, deletion of Gdt1p jeopardizes Ca^2+^ mediated signaling in yeast in response to salt stress (Demaegd et al., 2013). Gdt1 activity is negatively regulated by calcineurin, a Ca^2+^/calmodulin-activated Ser/Thr protein phosphatase (Snyder et al., 2017). The molecular mechanism(s) by which calcineurin regulates the function of Gdt1 and Vcx1 remain unknown, as neither protein undergoes changes in expression or mobility on SDS-PAGE upon activation/inhibition of calcineurin. In chondrocytes and in fibroblast cells of HsTMEM165 deficient patients TGF*β*/Smad2 and BMP signaling pathways are functionally impaired (Khan et al., 2021).

### TMEM165 role in pH homeostasis and trafficking-in glycosylation

TMEM165 is linked to glycosylation first by its transport activity. Many glycosyltransferases require Mn^2+^ as cofactor (i.e., *α*-1,3-*N*-acetylgalactosaminyltransferase, α-1,3-galactosyltransferase, β-1,3-glucuronosyltransferase and β-1,4-galactosyltransferase among others (Figure 5). Glycosylation defects might be attributed to disturbances in cation concentration balance, since Mn^2+^, Mg^2+^, Co^2+^, Zn^2+^, Ca^2+^, Fe^2+^, Cu^2+^, Cd^2+^, Ba^2+^, Ni^2+^ are cofactors of many glycosylation enzymes (Foulquier and Legrand, 2020). Interestingly, isogenic TMEM165 Knock Out human embryonic kidney cells, that are considered a CDG model system, were valuable in revealing that iron can rescue glycosylation defects (Vicogne et al., 2020).

Apart from modulating pH homeostasis, since cations taken up by acidic intracellular compartments are exchanged with H^+^ (Wang et al., 2020), TMEM165 can assure the elimination of proton overload generated by glycosylation and other metabolic processes. Recently, Zajac and collaborators (Zajac et al., 2024) provided an unequivocal demonstration of the importance of TMEM165 in intracellular pH dynamics by acting as a proton-activated, lysosomal Ca^2+^ importer in several cell types (i.e., *Saccharomyces cerevisiae* strain that lacks both the vacuolar Ca^2+^ importers, pmc1 and vcx1, and expresses HsTMEM165 and HeLa cells where HsTMEM165 was either knocked out or overexpressed). In these experiments the authors used complementary techniques to measure ion fluxes across intracellular biomembranes (ionic current recording and a pH-correctable, DNA-based fluorescent reporter for organellar calcium). Accordingly, it was also described that lysosomal H^+^ efflux through TMEM165 acidifies the perilysosomal area (maintaining its pH significantly below the value of the average cytosolic pH) in stably transfected HeLa cells with a constructed cDNA encoding for a fluorescent perilysosomal pH sensor targeted to the lysosomal surface, and a pH sensitive fluorescent protein, whose fluorescence intensity negatively correlates with the adjacent pH (Chen et al., 2024). This study also clearly showed that TMEM165 is required for the lysosomal H^+^ leakage in response to elevations in free cytosolic Ca^2+^ concentration, and its overexpression disturbs cellular pH homeostasis by causing acidification of the entire cytosol. Thereby, TMEM165 deficiency should be also consider in the context of membrane trafficking-related CDG.

Glycosylation is a complex sequential addition, binding and trimming of monosaccharides to decorate proteins with branched glycan structures. After synthesis, proteins must be transported through endoplasmic reticulum, different Golgi compartments and trans-Golgi network to be glycosylated and targeted to final destinations, e.g., the plasma membrane or organelles. Efficient protein glycosylation and trafficking require strict pH regulation in each Golgi compartment, as well as along secretion pathways. Certainly, dysregulation of pH homeostasis must contribute to defective protein glycosylation (Kellokumpu, 2019; Linders et al., 2020; D’Souza et al., 2021; Toustou et al., 2022).

## Predicting HsTMEM165 mutation-induced cardiomyopathies in CDG patients

### Vulnerability of TMEM165 functional domains

A cloud still hangs over the possibility of imprinting and epigenetic alterations of the HsTMEM165 gene and its intergenerational transmission. Conversely, occurrence of HsTMEM165 gene mutations in CDG patients are documented in the literature.

**Figure 10:**
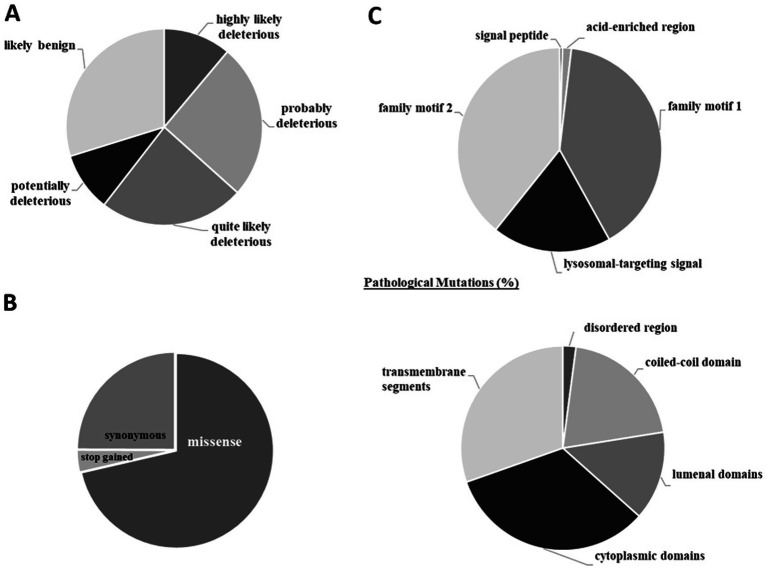
Human TMEM165 gene mutations confirmed at the protein level. **(A)** Deleteriousness of variants. **(B)** Types of gene mutations. **(C)** Localization of pathological mutations in structural and functional regions. Results are expressed in percentage of the total number of mutations. The CADD phred-like score was used to classify the variants according to their deleteriousness (<15.0 likely benign; 15.0–19.9 potentially deleterious; 20.0–24.9 quite likely deleterious; 25.0–29.9 probably deleterious; >29,9 highly likely deleterious). Data from CADD (Rentzsch et al., 2019) and ProtVar (Stephenson et al., 2024). Explanation in the text.

Of the more than 2,500 HsTMEM165 gene mutations confirmed at the protein level, only less than 30% are predictably benign (Figure 10A). This finding is not surprising, since the cellular functions of TMEM165 analyzed in the previous section allowed us to anticipate its broad physiological relevance. Most mutations described are of the missense type (~70%), while stop-gained ones represent less than 4% (Figure 10B). The introduction of a premature stop codon leads to render the protein non-functional and/or might contribute to generating transcript variants with altered functional properties.

Mutations have been described in all amino acid residues that make up the HsTMEM165 sequence. Those that have already gained recognition as pathogenic are found mainly in cytoplasmic domains (i.e., pathogenic mutations were found in all amino acids in the third short cytoplasmic domain) and transmembrane segments (except the fourth transmembrane segment where only pathogenic mutations were found in 24% of the amino acid residues) (Figure 10C). When considering the functional domains, pathogenic mutations are concentrated in the family motif 1 and 2, where all amino acid residues have at least one pathogenic mutation. Additionally, in the lysosomal-targeting signal 75% of mutated amino acid residues are associated with pathological conditions (Figure 10C). These findings are indicative of a profound impact on both cation/H^+^ antiport activity and subcellular distribution of HsTMEM165, the physiological relevance of which has been highlighted above.

Foulquier and collaborators (Foulquier et al., 2012) first demonstrated that HsTMEM165 deficiency impairs protein *N*-glycosylation. Subsequently, attention focused primarily on the functionality of HsTMEM165 mutants in the Golgi glycosylation, their expression levels, subcellular location, and targeting for lysosomal degradation in response to Mn^2+^ overload. The focus will now be on variant specific mechanisms of HsTMEM165 mutations and their downstream effects that impact cardiac function.

The substitution of the nucleotide cytosine in the glutamine codon CAG by thymine (151C > T) originates the amber codon (UAG). Consequently, the nonsense (NM_018475.5:c.151C > T) and the non-coding transcript variant (NR_073070.2:n.384C > T) have been included in the ClinVar database associated with ADRB1 polymorphism. ADRB1 gene encodes the *β*_1_-adrenergic receptor (P08588), a glycoprotein (*N*-linked GlcNAc in position 15) (Meijer et al., 2015). This receptor is the dominant β-adrenergic receptor in the heart that when activated increases sinoatrial nodal, atrioventricular nodal, and ventricular muscular firing leading to increased heart rate (chronotropy), increased contractility (inotropy) and increased relaxation of the heart muscle (lusitropy) (Chevalier et al., 2023).

The missense mutation Glu108 > Gly (gAa/gGa) in the family motif 1 (E108LGDKT113) was shown by Schulte and collaborators in 2016 (Schulte Althoff et al., 2016) The replacement of the medium size and acidic glutamate by glycine causes glycosylation defects and reduces the capacity of the mutated protein to transport Ca^2+^ and Mn^2+^ without altering the affinity for these cations (Schulte Althoff et al., 2016; Stribny et al., 2020). Glycosylation defects observed in TMEM165-knockdown HeLa cells were only partially restored by expression of the HsTMEM-Glu108 > Gly, which was properly localized in the Golgi apparatus (Potelle et al., 2017; Lebredonchel et al., 2019a) and partially resistant to Mn^2+^ induced lysosomal degradation (Lebredonchel et al., 2019a). Sequence analysis of HsTMEM165 in a patient with cardiac dysfunction showed homozygosity for the missense mutation c.323 A > G in exon 2 (Schulte Althoff et al., 2016). The same mutation was present in a heterozygous state in both parents. Accordingly, missense mutations of the other evolutionary conserved amino acids residues in the family motif 1, namely the negatively charge Asp111, the large positively charges Lys112 and the polar Thr113, as well as the neighbor large, hydrophobic, aromatic Phe114 by the small aliphatic glycine, impaired the glycosylation of the lysosome-associated membrane glycoprotein 2 (LAMP2) (Lebredonchel et al., 2019a). Notably, TMEM165 deficiency leads to a defect in LAMP2 glycosylation characterized by an abnormal, partially glycosylated form (Vicogne et al., 2020; Durin et al., 2022). LAMP2 is the gene defective in Danon disease, an X-linked lysosomal storage disorder characterized by severe cardiomyopathy, vacuolar myopathy and intellectual deficit (Endo et al., 2015; Sugie and Nishino, 2024). Danon disease is caused by loss of LAMP2 itself, rather than a glycosylation defect, but the observed LAMP2 underglycosylation in TMEM165-depleted cells (Foulquier et al., 2012) suggests an avenue by which TMEM165 dysfunction might indirectly affect lysosomal proteins relevant to cardiac pathology. These symptoms are shared by several heart congenital diseases, such as myofibrillar myopathy 4, dilated cardiomyopathy 1X and 2F, as well as early-onset myopathy with fatal cardiomyopathy, whose causal genes are LDB3, FKTN, BAG5 and TTN, respectively. Mutations in LDB3 gene have been also associated with muscular dystrophy (Selcen and Engel, 2005) and several cardiomyopathies (arrhythmogenic right ventricular, noncompaction and dilated cardiomyopathies) (Vatta et al., 2003; Arimura et al., 2004; Xi et al., 2012; Lopez-Ayala et al., 2014). LDB3 gene encodes the LIM domain-binding protein 3, also known as Z-band alternatively spliced PDZ-motif protein, a Zn^2+^ binding Z-line protein of the sarcomere (Faulkner et al., 1999). FKTN gene encodes the single-pass type II membrane protein of the Golgi apparatus, fukutin (ribitol-5-phosphate transferase FKT). The enzyme catalyzes the first step in the formation of the ribitol 5-phosphate tandem repeat, which links the phosphorylated *O*-mannosyl trisaccharide to the ligand binding moiety composed of repeats of 3-xylosyl-*α*-1,3-glucuronic acid-β-1. In MCK-Fktn-cKO mice, acute fukutin elimination caused severe cardiac dysfunction and accelerated mortality with myocyte contractile dysfunction and disordered Golgi-microtubule networks (Ujihara et al., 2019). This study highlighted the crucial role of protein glycosylation in maintaining myocyte physiology and thus preventing heart failure. The BAG5 chaperone improves cardiomyocyte viability and has a protective role during cardiac stress due to calcium depletion, ischemia and oxidative stress that causes accumulation of defective proteins in the endoplasmic reticulum (Gupta et al., 2016). TTN gene encodes the essential component of cardiac muscle sarcomeres titin. It is a nonspecific serine/threonine protein kinase that requires divalent cations (Ca^2+^, Mg^2+^, Mn^2+^ and/or Zn^2+^) and calmodulin to full activation (Stroik et al., 2024).

Amino acid residues located at the transition between the first transmembrane segment and the cytosol domain make up the lysosomal targeting signal, Y124NRL127. Pathogenic mutations in the lysosomal targeting signal disrupt exit from Golgi apparatus, delay clathrin-mediated endocytosis and perturb endo-lysosomal ion homeostasis, altering ion handling in cells. The mutation Tyr124 > Ser (uAu > uCu) and Tyr124 > Phe (uAu/uUu), and eventually Tyr124 > Asp (Uau/Gau), mainly affect the subcellular location of HsTMEM165. Apparently, these mutants are unable to exit from the Golgi apparatus (Rosnoblet et al., 2013). The wild-type HsTMEM165 when expressed in Gdt1 deficient yeast was present within Golgi compartment, plasma membrane and late endosomes/lysosomes. The mutant Tyr124 > Ser accumulated essentially in the Golgi compartment and was undetectable at the plasma membrane, while expression of the mutant Tyr124 > Phe was reduced in Gdt1 deficient yeast considerably. Arginine at position 126 is phylogenetically strictly conserved, and all possible single nucleotide variants (Arg126 > Ser (Cgc/Agc), Arg126 > Cys (Cgc/Ugc), Arg126 > Gly (Cgc/Ggc), Arg126 > His (cGc/cAc), Arg126 > Pro (cGc/cCc) and Arg126 > Leu (cGc/cUc)) are pathogenic mutants (Sparks and Krasnewich, 2005; Foulquier et al., 2012; Demaegd et al., 2013; Rosnoblet et al., 2013; Schulte Althoff et al., 2016; Colinet et al., 2017; Zajac et al., 2024). These mutations delay the internalization of HsTMEM165 by clathrin-mediated endocytosis (Rosnoblet et al., 2013). Thus, it was demonstrated in fibroblasts isolated from CDG patients and by the expression of natural human variants in lci-1 +/− worms, that in addition to anomalous intracellular localizations, ionic homeostasis was also altered, mainly in lysosomes and endosomes (Demaegd et al., 2013; Zalac et al., 2024). The subcellular location of TMEM165 was also challenged by Lebredonchel and collaborators (Lebredonchel et al., 2019a) in control and KO TMEM165 HEK293T cells using mutated TMEM165 plasmids. This study showed that arginine, a large positively charged amino acid residue at positions 198–200 and 208–210 of the large cytoplasmic domains (Arg173-Lys228), is not involved in the protein expression, neither in its Golgi localization nor in Mn^2+^-induced degradation of TMEM165. Arginine mutations at position 126 of the HsTMEM165 polypeptide chain have been reported in homozygous and heterozygous CDG patients accompanied by family history (Foulquier et al., 2012; Rosnoblet et al., 2013). Interestingly, substitution of the large amino acid leucine for glycine at position 127 of the TMEM165 polypeptide chain had no effect on subcellular localization (Rosnoblet et al., 2013). Conversely, this mutation decreases the Ca^2+^ affinity of TMEM165 during cation uptake into the Golgi apparatus without affecting the affinity for Mn^2+^. However, this effect was not confirmed when the kinetic analysis of TMEM165 transport activity was evaluated in *Lactococcus lactis* expressing the wild-type and mutated variants (Stribny et al., 2020).

Natural mutations in familial motif 2 (E248WGDRS253) reported in CDG patients appear particularly multifaceted. The overexpression of the mutant Glu248 > Ala (gAa/gCa) heavily compromises Ca^2+^ transport in HeLa cells (Zajac et al., 2024). Glu248 also appears to be crucial for protein glycosylation, since the mutant Glu248 > Gly (gAa/gGa) is unable to restore LAMP2 glycosylation (Lebredonchel et al., 2019b; Lebredonchel et al., 2019a). Replacement of Asp251, Arg252, Ser253 and Gln254 with glycine produced heterogeneous results with respect to subcellular mislocalization and sensitivity to high Mn^2+^-induced lysosomal degradation. Glycine at position 304 in the sixth transmembrane segment is phylogenetically strictly conserved. In a patient compound heterozygous the mutant HsTMEM165 (304Gly > Arg) was detected (Rosnoblet et al., 2013). This missense mutation did not alter the localization of TMEM165 in the Golgi apparatus, but the mutated TMEM165 had a decreased affinity for Ca^2+^ and maintained that for Mn^2+^ unchanged (Foulquier et al., 2012; Zajac et al., 2024). Therefore, it caused acidification in lysosomal and endosomal compartments in fibroblasts isolated from HsTMEM165-deficient patients (Demaegd et al., 2013).

### Impact of TMEM165 and glycome on Ca^2+^ handling and ion channel activity

Throughout the text, several clues were provided as to how HsTMEM165 may be important for the contractile activity of cardiomyocytes and for the onset of cardiomyopathies associated with its pathogenic mutations. More importantly, TMEM165 is the only high-capacity, low-selectivity Ca^2+^/H^+^ antiporter in acidic organelles of mammalian cells. Therefore, it should play a fundamental role in the complex machinery that assures Ca^2+^ handling in cardiomyocytes and contractility. Internal Ca^2+^ stores from a phylogenetically wide range of different organisms can be also mobilized by cyclic ADP-ribose (cADPR) and nicotinic acid adenine dinucleotide phosphate (NAADP), in addition to the major D-myo-inositol 1,4,5-trisphosphate (IP_3_)-mobilizable Ca^2+^ pool (Lee, 1997; Lee, 2000; Walseth and Guse, 2021). These Ca^2+^ mobilizing agents are closely related, since the same metabolic enzyme ADP-ribosyl cyclase/cyclic ADP-ribose hydrolase 1, can, under appropriate conditions, synthesize either of them. This suggests that a unified mechanism may regulate both pathways. Interestingly, the enzyme contains at least three *N*-glycosites (Chen et al., 2009) and NAADP production is much higher at pH 5.0 than 7.4 (Moreschi et al., 2006). NAADP induces Ca^2+^ release from endoplasmic reticulum in cardiomyocytes (Yusufi et al., 2001). In turn, this is inhibited by L-type calcium channel blockers and bafilomycin A1, a selective V-type H^+^-ATPase inhibitor, whereas it is not affected by inhibitors of IP_3_ or ryanodine channels. The three Ca^2+^-mobilizing second messengers, IP_3_, cADPR and NAADP, are simultaneously involved in the generation and shaping of intracellular Ca^2+^ signaling (da Silva and Guse, 2000; Lee, 2001; Walseth and Guse, 2021). This feature reflects the need for strict control of the spatial and temporal propagation of the intracellular Ca^2+^ wave to give the appropriate physiological response to different extracellular signals. Ca_V_1.2 L-type calcium channels in cardiomyocytes are essential for normal cardiac function, playing a crucial role in excitation-contraction coupling and action potential duration. These channels mediate Ca^2+^ influx, initiating the intracellular Ca^2+^ transient ensured by their intracellular reservoirs, including HsTMEM165, which ultimately leads to muscle contraction and subsequent relaxation (Terrar, 2022). Furthermore, the surface expression and function of Ca_V_1.2 subunits are modified by *N*-glycosylation (Park et al., 2015).

TMEM165 deficiency perturbs Golgi Mn^2+^/Ca^2+^ homeostasis and broadly disrupts glycosylation which is a key step in the metabolism of proteoglycans, glycolipids and gangliosides (Lebredonchel et al., 2019b; Lebredonchel et al., 2019a; Potelle et al., 2016; Tian et al., 2018; Grewal et al., 2021; Rini et al., 2022; Dei Cas et al., 2023; Jankauskas et al., 2024). Hypoglycosylation reasonably perturbs neuronal excitability and seizure threshold, which are observed in CDG patients (Scott and Panin, 2014). *N*-glycosylation modulates trafficking and gating of voltage-gated Na^+^, K^+^ and Ca^2+^ channels, as well as and ligand-gated receptors of the most abundant excitatory and inhibitory neurotransmitters in the brain, namely glutamate (AMPA and NMDA receptors) and *γ*-aminobutyric acid (GABA_A_ receptor) (Ednie et al., 2015; Skrenkova et al., 2018; Kandel et al., 2018). Moreover, TMEM165 deficiency also disrupts, in a Mn^2+^-dependent manner, the biosynthesis of essential glycosaminoglycans (heparan sulfate and chondroitin sulfate), causing perineuronal net dysfunction that likely contributes to abnormal excitability and cognitive deficits (Scott and Panin, 2014; Skog et al., 2016; Sorg et al., 2016; Benner et al., 2023; Auer et al., 2025). Hypogalactosylation of glycosphingolipids, particularly of gangliosides that account for 80% of all glycans and >75% of the sialic acid present in the brain (Sipione et al., 2020), will certainly impact on the organization of the nodes of Ranvier and myelin sheaths, that are of paramount importance for action potential conduction and white-matter development (Susuki et al., 2007; Briggs and Hohenester, 2018; McGonigal and Willison, 2022).

Recently, reference structure libraries of the human cardiac glycome containing 265 *N*- and *O*-glycans were generated by porous graphitized carbon (PGC) LC interfaced with MS (PGC-LC–MS) (Ashwood et al., 2020). The cell type specific cardiomyocyte *N*-glycome is enriched in high mannose structures and remodels markedly during differentiation, whereas the *O*-glycan profile remains almost unchanged (Ashwood et al., 2020). Protein glycosylation pathways contribute to define how action potentials are propagated in the heart, since calcium, potassium and sodium ion channels are cell surface glycoproteins. For example, how blocking the binding of the *N*-glycan to the *β* subunit of the type I transmembrane K^+^ channel KCNE1 prevents its correct insertion into the membrane to form functional K^+^ channels that produce the slowly activating cardiac Iks current has been masterfully demonstrated (Bas et al., 2011).

### Cardiac findings in TMEM165-CDG case reports

It is expected that there will be a permanent increase in experimental evidence supporting that mutations in the HsTMEM165 gene are the etiological cause of many congenital multisystemic diseases and syndromes. Of great importance is the fact that the mutated HsTMEM165 sequences available in ClinVar are of germline origin, meaning that they can be transmitted to offspring.

Mutations found in CDG patients, whose symptoms and family history have been well documented are now spotlighted. Two children of consanguineous parents were born with HsTMEM165 deficiency, the first died at 5 months of age and the other was born with heart defects. Echocardiography revealed a small apical ventricular septal defect, a patent foramen oval and a small patent ductus arterior with mild signs of right ventricular hypertrophy (Schulte Althoff et al., 2016). This is a particularly well documented clinical case, since a sibling already diagnosed with the disease allowed prenatal diagnosis and follow up starting from birth. Only 3 min after birth, an abnormal glycosylation pattern was already observed characterized by increased amounts of tri-, di-, and monosialotransferrin, with tetra- and trisialotransferrin still forming the largest fractions. Over the next few weeks, the spectrum of transferrin changed to less sialylated and then also to hypogalactosylated forms, resulting in the nearly complete loss of tetrasialotransferrin. From week 9 forward, galactose and sialic acid residues were missing in equal proportions, indicating that galactosylation was compromised. *O*-Glycosylation was also impaired right from birth with patterns deteriorating over time until the patient died due to nephrotic syndrome. Cases of heart defects reported in TMEM165-CDG patients range from severe and fatal, as previously described, to moderate and minor alterations or even undetectable ones due to premature death (Gehrmann et al., 2003). A patient with a homozygous deep intronic mutation (Gly182 > Ala) in HsTMEM165, with symptoms of CDG with a serum transferrin type 2 pattern and a cathodal shift of serum apolipoprotein C-III, at 14 months of age had normal cardiac morphology and function except for a small pericardial effusion visible on echocardiography (Zeevaert et al., 2013).

## Where to slot TMEM165 in today’s diagnostics?

Specific treatments are available or are under clinical investigation for the TMEM165-CDG. At the molecular level, patients with TMEM165-CDG present altered protein glycosylation profiles, mainly in terms of hyposialylated and hypogalactosylated *N*-glycans (Foulquier et al., 2012; Potelle et al., 2016). The strong protein hypogalactosylation in these patients has been compensated with oral galactose supplementation (1 g galactose/kg/d) that improved the clinical and biochemical parameters (Morelle et al., 2017). Glycosylation abnormalities were reversed by exposure to low Mn^2+^ without or with D-galactose (Vicogne et al., 2020; Durin et al., 2024). Sucessuful rescue of *N*-glycosylation in TMEM165 knockout HEK293 cells has been achieved by extracellular administration of 1 μM MnCl_2_ (Houdou et al., 2019). Given Mn^2+^ neurotoxicity, any Mn^2+^-based intervention requires protocolized monitoring (blood Mn, T1-weighted MRI signal in the globus pallidus, neurologic exams, etc) and explicit stopping rules (Majewski et al., 2024). Model systems, particularly the patient-derived ones, provide a coherent mechanistic basis for *in vitro* diagnostics and diagnostic targeting. Accurate disease detection, early intervention and personalized treatments are fundamental to personalized medicine, which aims to provide the most appropriate and timely treatment for each patient to produce more effective results and minimize unwanted side effects. Alternatively, antisense RNA therapy targeting TMEM165 mRNAs has been valued as an alternative patient-centered therapeutic strategy. For instance, in one case of pathological deep intronic variant, successful alternative splicing of the abnormal pseudoexon was achieved through therapy with antisense morphonucleotide oligonucleotides targeting TMEM165 mRNA, which restored normal protein levels in the Golgi apparatus of patient-derived fibroblasts (Yuste-Checa et al., 2015). Compared to mRNA technologies, circRNA-based ones have greater potential for therapeutic application in monogenic diseases. The resistance of circRNA to degradation and its multifaceted biological capacity allows it to act both as a protein-coding molecule and as a non-coding regulator. Despite the undeniable interest in applying circRNA in the context of TMEM165 with cardiac manifestations (Li et al., 2022; Liu et al., 2024), gene and cell therapies remain a challenge to this day.

Traditionally, the diagnosis of CDG begins with screening blood tests followed by additional biochemical tests. Molecular genetic testing to identify the specific genetic mutation is performed as a final step, allowing for a definitive and reliable diagnosis. In most cases, this strategy involves a prolonged time between the onset of symptoms, suspicion, and diagnosis, with significant negative impacts on patient healthcare (Granjo et al., 2024). Due to the diversity of symptoms and the degree of severity, the diagnosis of the multisystem TMEM165-CDG is still not an easy task. Usually, serum transferrin isoform analysis by isoelectric focusing, capillary electrophoresis or high-performance liquid chromatography is the front-line biochemical screen to detect *N*-glycosylation pattern of transferrin revealing abnormal protein glycosylation (Schulte Althoff et al., 2016; Raynor et al., 2024; Raynor et al., 2025; Hall et al., 2025).

Despite the accumulated experience in performing this routine clinical analysis, it can lead to diagnostic pitfalls, due to inter-laboratory variability, phenotypic variability, age effects and biochemical mimicry (Raynor et al., 2025; Raynor et al., 2024; Hall et al., 2025). For instance, disorders of systemic manganese homeostasis can yield similar under-galactosylation signatures characteristic of post-synthetic processing defects. Moreover, TMEM165-CDG may evolve toward less sialylated/galactosylated isoforms over the first weeks of live (Schulte Althoff et al., 2016; Raynor et al., 2024; Raynor et al., 2025; Hall et al., 2025). Therefore, normal neonatal or single early transferrin and glycan profiles do not exclude TMEM165-CDG. Consequently, good practices usually recommend overtime repeats including biochemical analysis of other circulating and cellular biomarkers and/or broaden to glycomic (Abu Bakar et al., 2018). Analysis of blood apolipoprotein CIII (Wada et al., 2022), high-resolution screening of urine oligosaccharides by MALDI-TOF MS (Pino et al., 2025) and the cellular biomarkers lysosomal associated membrane protein 2 (LAMP2; Morelle et al., 2017) are used to narrow down specific glycosylation defects. It is worth highlighting that the significant pattern of LAMP2 hypoglycosylation in TMEM165-CDG cells (Morelle et al., 2017) was easily detected by Western blot analysis in TMEM165 mutant cells (Houdou et al., 2019). Mass spectrometry, by revealing the structures of glycans bound to proteins (glycoproteomics) or as free structures (glycomics), should be a clinically relevant first-line screening technique for defining the characteristic subgalactosylation signature of the TMEM165 defects (Abu Bakar et al., 2018; Raynor et al., 2024; Raynor et al., 2025).

Global multi-omics that contributes to the mechanistic understanding of diseases and assures the identification of new biomarkers, is crucial to guide clinical surveillance. It offers a better tracking of disease progression, biomarker discovery and novel therapies. Large-scale studies of proteins and glycans, focusing on structure, function and interactions, are scarce. Particularly, those focusing on CDGs and cardiomyopathies using tissue and blood samples of patients stand out (Captur et al., 2020; Xie et al., 2021; Cirnigliaro et al., 2022). For instance, myocardial and extracellular matrix proteo/glycoproteomics could map defects in Mn^2+^-dependent glycosyltransferase in sarcomeric glycosylation, cardiac extracellular matrix, and ion channels, elucidating cardiac vulnerability and providing information on biomarkers. The metabolomics approach, by highlighting specific metabolic alterations and dysregulation of biochemical pathways, is essential for elucidating the pathophysiological and biochemical mechanisms underlying CDG. With the development of powerful high-throughput and high-speed technologies, like next-generation sequencing (NGS) and whole-genome sequencing (WGS), that provide the complete set of DNA including the interactions of all genes with each other and the environment, genomics is of utmost importance to personalized medicine. These advances in genomics should encourage a definitive improvement in the diagnostic workflow for patients, considering molecular genetic testing as the initial step in the TMEM165-CDG diagnostic paradigm during routine clinical practice. There is an urgent need to generalize the adoption of multidisciplinary approaches that simultaneously encompass clinical evaluation and multiomics analysis, since initiating the most appropriate treatment early is generally associated with better health outcomes. Because it is a potentially very serious hereditary disease, TMEM165-CDG needs to be detected quickly to limit diagnostic hesitancy and introduce curative treatment (when available) and/or symptomatic supportive therapies as soon as possible (Foulquier et al., 2012; Raynor et al., 2024; Raynor et al., 2025; Hall et al., 2025). Furthermore, the rapid identification of an index patient with CDG allows for prenatal diagnosis for future at-risk pregnancies in the related family (Greczan et al., 2022).

Meanwhile, experimental validation of predictive models remains the immediate priority to clarify many open questions central to understanding the physiological and pathophysiological role of TMEM165. For instance, (1) What is the precise stoichiometry/selectivity of Ca^2+^ versus Mn^2+^ under pH fluctuations? (2) How does isoform-specific N-terminal regulation (phosphorylation versus glycosylation) gates activity and trafficking? (3) Does organ-specific expression of short isoforms act as local ER/Golgi shunts? (4) What is the best therapeutic strategy to normalize glycosylation in TMEM165-CDG, galactose/ion supplementation, ion pump modulation, or proteostasis?

## Conclusion

**Figure 11:**
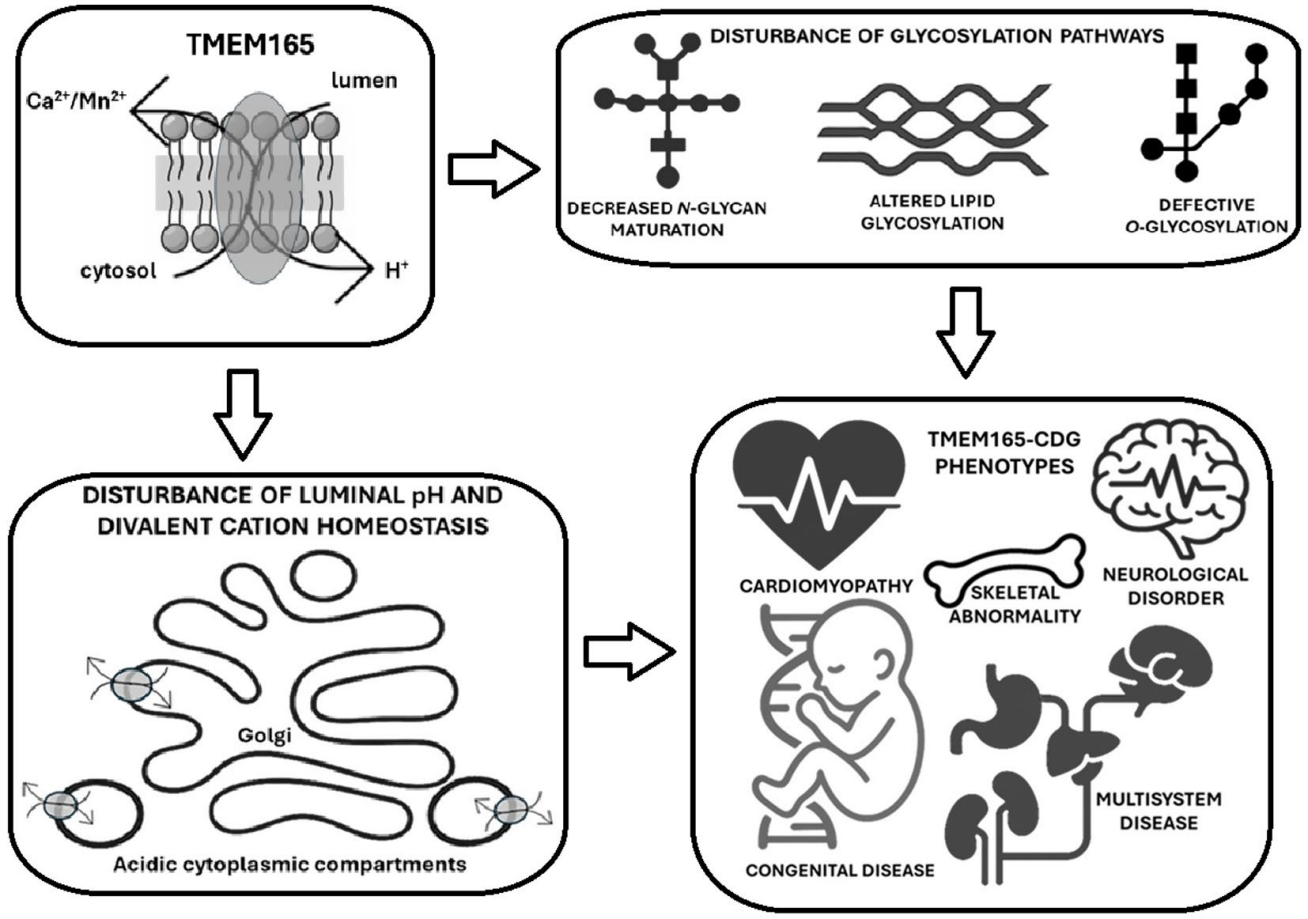
Schematic representation of TMEM165’s molecular function, its impact on glycosylation pathways, and the downstream effects that could link TMEM165 dysfunction to cardiac and neurological pathologies. The main concepts of each section are visually interconnected to serve as a roadmap of how TMEM165 deficiencies can lead to multisystemic clinical manifestations, emphasizing congenital cardiomyopathies.

TMEM165 is established as a protein associated with protein glycosylation and of paramount importance for the maintenance of the structural and functional integrity of the endoplasmic reticulum, Golgi apparatus, trans-Golgi network, cytoplasmic acidic vesicles, as well as pH and cation homeostasis in human cells. TMEM165 accelerates the recovery of cells from cytosolic cation overload and alkalinization. Structural and functional interactions between proteins and their domains underlie nearly all biological processes. To be biologically sensible, computational predictions must be tested experimentally. In summary, experimental validation is needed to confirm many of the physiological and pathophysiological implications of the predicted possibility of regulating the subcellular distribution and activity of isoforms of TMEM165. Figure 11 shows a schematic representation of TMEM165’s molecular function, its impact on glycosylation pathways, and the downstream effects that could link TMEM165 dysfunction to cardiac and neurological pathologies.

Cardiac-related problems in CDG patients are often fatal and therefore no less important than more common clinical symptoms, e.g., neurological and musculoskeletal symptoms. Notably, cardiac functional abnormalities are observed in roughly 20% of reported CDG cases - a significant minority that suggests such complications may be underrecognized. Therefore, it is worthwhile to screen for underlying cardiomyopathy in CDG patients of unknown etiology and, conversely, to consider screening for CDG of unknown etiology in patients with unexplained cardiomyopathy. Seemingly CDG are often monogenic diseases, e.g., TMEM165-CDG. Genetic diagnosis based on a causal gene pinpoints the specific gene mutation that directly causes the disease. Therefore, it is crucial for accurate diagnosis of comorbidities in other organ systems and selection of appropriate therapies.
