# Supplementary material for: Putative role of TMEM165 in congenital cardiomyopathies
Source: Front Mol Neurosci. 2026 Jan 9;18:1692968. doi: 10.3389/fnmol.2025.1692968 (PMC12827658; doi:10.3389/fnmol.2025.1692968)
Supplement: Supplementary file 3 [file Table_3.docx]

**Supplemental Table 3S. Human TMEM165 interatom.**

| Gene | Protein | Interaction type | | Combined confidence | Co-expression score |
| --- | --- | --- | --- | --- | --- |
| **Experimental Evidence** | | | | | |
| ATP2C1 | Calcium-transporting ATPase type 2C member 1 | Functional | | 0.718 | 0.097 |
| ATP2A3 | Sarcoplasmic/endoplasmic reticulum calcium ATPase 3 | Functional in other organisms | | 0.469 | 0.042 |
| ATP2A2 | Sarcoplasmic/endoplasmic reticulum calcium ATPase 2 | Functional in other organisms | | 0.449 | 0.060 |
| **Inferred from Sequences** | | | | | |
| TM9SF2 | Transmembrane 9 superfamily member 2 | Functional | | 0.625 | 0.086 |
| SRD5A3 | Polyprenol reductase | Functional | | 0.692 | 0.060 |
| ALG2 | Alpha-1,3/1,6-mannosyltransferase | Functional | | 0.590 | 0.060 |
| GOSR1 | Golgi SNAP receptor complex member 1 | Functional | | 0.586 | 0.082 |
| SLC10A7 | Sodium/bile acid cotransporter 7 | Functional | | 0.562 | 0.049 |
| UNC50 | Protein unc-50 homolog | Functional | | 0.554 | 0.142 |
| PIGO | GPI ethanolamine phosphate transferase 3 | Functional | | 0.522 | 0.060 |
| GOLIM4 | Golgi integral membrane protein 4 | Functional | | 0.534 | 0.056 |
| **Other** **Predictable Structural/Functional Interactions** | | | | | |
| MAPK6 | Mitogen-Activated Protein Kinase 6 | | | | |
| TRIM25 | E3 ubiquitin/ISG15 ligase TRIM25 | | | | |
| CUL3 | Cullin-3 | | | | |
| Total | 17 |  |  | |  |

Source: STRING, ComPPI and TFLink databases [Veres et al., 2015; Szklarczyk et al., 2021; Liska et al., 2022].
